# Evaluating Large Language Model–Supported Instructions for Medication Use: First Steps Toward a Comprehensive Model

**DOI:** 10.1016/j.mcpdig.2024.09.006

**Published:** 2024-12

**Authors:** Zilma Silveira Nogueira Reis, Adriana Silvina Pagano, Isaias Jose Ramos de Oliveira, Cristiane dos Santos Dias, Eura Martins Lage, Erico Franco Mineiro, Glaucia Miranda Varella Pereira, Igor de Carvalho Gomes, Vinicius Araujo Basilio, Ricardo João Cruz-Correia, Davi dos Reis de Jesus, Antônio Pereira de Souza Júnior, Leonardo Chaves Dutra da Rocha

**Affiliations:** a Health Informatics Center, Faculty of Medicine, Universidade Federal de Minas Gerais, Belo Horizonte, Minas Gerais, Brazil; b Arts Faculty, Universidade Federal de Minas Gerais, Belo Horizonte, Minas Gerais, Brazil; c Department of Pediatrics, Faculty of Medicine, Universidade Federal de Minas Gerais, Belo Horizonte, Minas Gerais, Brazil; d Department of Design Technology, School of Architecture, Universidade Federal de Minas Gerais, Belo Horizonte, Minas Gerais, Brazil; e Department of Obstetrics and Gynecology, Faculty of Medical Sciences, Universidade Estadual de Campinas, Campinas, São Paulo, Brazil; f Departamento de Medicina da Comunidade Informação e Decisão em Saúde, National Secretary of Primary Care of the Brazilian Ministry of Health, Brasília, Brazil; g MEDCIDS, Porto University, Porto, Portugal; h Department of Computer Science, Universidade Federal de São João Del Rei, São João del Rei, Minas Gerais, Brazil

## Abstract

**Objective:**

To assess the support of large language models (LLMs) in generating clearer and more personalized medication instructions to enhance e-prescription.

**Patients and Methods:**

We established patient-centered guidelines for adequate, acceptable, and personalized directions to enhance e-prescription. A dataset comprising 104 outpatient scenarios, with an array of medications, administration routes, and patient conditions, was developed following the Brazilian national e-prescribing standard. Three prompts were submitted to a closed-source LLM. The first prompt involved a generic command, the second one was calibrated for content enhancement and personalization, and the third one requested bias mitigation. The third prompt was submitted to an open-source LLM. Outputs were assessed using automated metrics and human evaluation. We conducted the study between March 1, 2024 and September 10, 2024.

**Results:**

Adequacy scores of our closed-source LLM’s output showed the third prompt outperforming the first and second one. Full and partial acceptability was achieved in 94.3% of texts with the third prompt. Personalization was rated highly, especially with the second and third prompts. The 2 LLMs showed similar adequacy results. Lack of scientific evidence and factual errors were infrequent and unrelated to a particular prompt or LLM. The frequency of hallucinations was different for each LLM and concerned prescriptions issued upon symptom manifestation and medications requiring dosage adjustment or involving intermittent use. Gender bias was found in our closed-source LLM’s output for the first and second prompts, with the third one being bias-free. The second LLM’s output was bias-free.

**Conclusion:**

This study demonstrates the potential of LLM-supported generation to produce prescription directions and improve communication between health professionals and patients within the e-prescribing system.

Large language models (LLMs) are reshaping the landscape of artificial intelligence (AI) applications, namely in the domain of health communication.^1^ A critical aspect of LLMs’ deployment involves prompt design—strategically developing commands to optimize a model’s output for specific tasks.^2^ Even in their untrained state, LLMs are capable of responding to queries presented in free-text format. However, when sufficiently trained, these models have significant potential to foster the quality of health assistance.^3^

e-Prescribing systems exemplify the integration of digital technology and health care, providing clear, legible prescriptions directly to pharmacies and mitigating the risks associated with unclear, handwritten medical commands.^4^ These systems not only streamline the process of medication dispensation but also incorporate standards and legal requirements to ensure safety and compliance with pharmacy prescription requirements.^5^ Despite these advances, medication nonadherence and unsafe use remain a challenge for national health systems, resulting in avoidable spending, health impact, and economic burdens.^6^ Lack of clarity in the language of prescriptions can significantly impact their readability and comprehension, potentially confusing patients about the proper use of their medications.^7^ Several studies have highlighted the inadequacy of current written patient information in prescriptions in effectively communicating important drug risks of unsafe usage.^8^, ^9^, ^10^ Additionally, there are also issues with prescriber behavior and fragmented medication use data, highlighting the need for further enhancements.^11^ Beyond standardizing prescriptions, providing personalized instructions represents a critical gap in current systems, meriting targeted improvements. Such enhancements should consider individual patient factors, including educational level, age, gender, and lay persons’ need for explanations that avoid medical jargon. Recognizing each patient’s unique characteristics can improve the safe use of medications, which is crucial to minimize incidents arising from misuse.^4^ This is in line with the tenets of health literacy, defined by the World Health Organisation as people’s ability to “gain access to, understand and use information in ways which promote and maintain good health for themselves, their families and their communities.”^12^ A review regarding the feasibility and impact of AI on pharmacy reported the potential of AI to predict response to treatments, adverse effects, and treatment adherence, resulting in more support to prescribers, providers, and patients.^13^

Given the present e-prescribing limitations and the potential of AI support for patient care, this study explores the use of LLMs, GPT-4 (OpenAI) and Llama 3 (Meta) to improve the clarity and personalization of medication directions for future use in e-prescribing platforms. This approach does not aim to replace e-prescribing but rather to augment it, drawing on a simulated primary care ambulatory setting and offering more tailored and comprehensible instructions for medication use, thereby potentially reducing errors and improving treatment outcomes.

## Patients and Methods

We prioritized prompt design to tailor GPT-4 and Llama 3 (Meta-Llama 3 8B) models to the specific needs of health professionals, providing clear and appropriate guidance for medication use alongside e-prescriptions. We started with GPT-4, a closed-source model widely tested and reported on for health applications, and then used an open-source one, Llama 3.^14^ To avoid hallucinations, we set the parameter temperature to 0 for GPT-4 and 0.2 for Llama 3.

**Figure 1:**
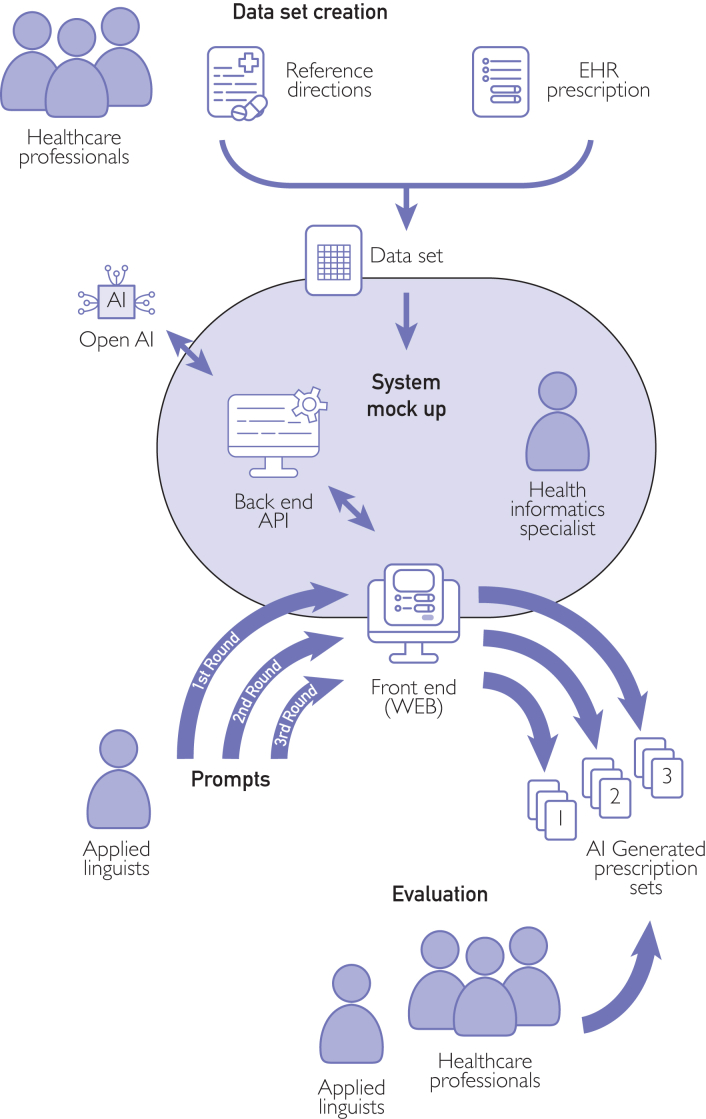
Diagram flow of our large language model–supported instruction generation. AI, artificial intelligence; API, application program interface; EHR, electronic health record.

The process involved defining a set of guidelines that reflect the individuality of each patient, thereby enhancing the overall prescribing process. In response to the need for curated standard requirements for patient medication instructions, we carried out a search that yielded 42 reports, pamphlets, and booklets about best practices for the safe use of medication. The list identifying these reports can be found in Supplemental File 1 (available online at http://www.mcpiqojournal.org). Besides medical guidance, we prioritized guidelines for clarity, acceptability, and personalization of texts to encourage patient adherence. These requirements included considering the context in which terms and expressions are used and ensuring that they are appropriate and respectful within the specific cultural context and free of gender, ethnicity, age group, culture, or ability bias. This set of recommendations is registered at Protocols.IO in the original Portuguese version.^15^ This foundational step served both as a benchmark for calibrating GPT-4 and Llama 3 prompts and as a standard for human validation of each round. The diagram in Figure 1 shows the conceptual flow of using LLMs in our electronic Sistema Único de Saúde (e-SUS) Primary Care e-prescription system. We started the study in March 1, 2024 and concluded in September 11, 2024.

### Simulated Scenario Dataset

**Figure 2:**
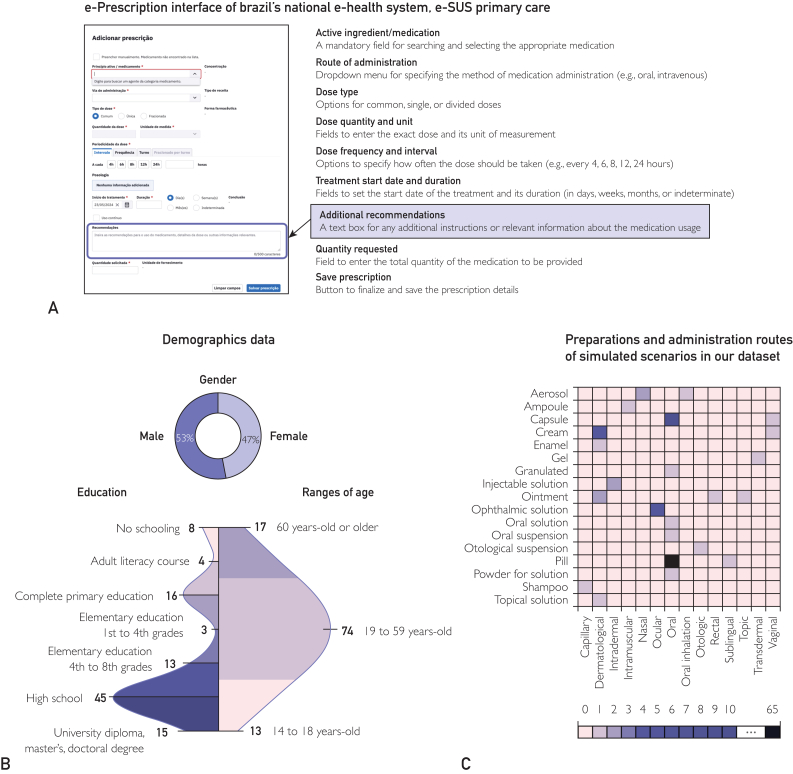
Source of variables for simulated scenarios and characterization of the dataset (n=104).

The dataset consists of simulated scenarios comprising a medication, its posology as prescribed by a health care professional, and personas with particulars of gender, age, and education who are the target of the instructions to be generated. These scenarios were collaboratively developed by a multidisciplinary team comprising health care professionals, health informatics specialists, pharmacists, and applied linguists. Medication prescriptions were modeled within Brazil’s national electronic health record (EHR) system for primary health care, the e-SUS Primary Care. The system provides prescribers full access to the patient’s medication history, allowing them to prescribe specific medications by selecting drug label, concentration, dosage, administration route, frequency of use, and quantity from structured dropdown menus. Figure 2A shows a screenshot of the e-prescription interface of Brazil’s Unified Health System, e-SUS Primary Care. The LLM-generated instructions are intended to support the prescriber in filling in “Additional Recommendations,” an optional free-text field in the EHR form (highlighted in Figure 2A). The system interface is designed to enhance the accuracy and clarity of medication prescriptions. The dataset, detailed in Supplemental File 2 (available online at http://www.mcpiqojournal.org), was tailored to reflect the clinical practices of physicians on our team, with each scenario being carefully constructed to account for a patient’s educational background, gender, age, and specific treatment indications based on the International Classification of Primary Care, 2nd edition (ICPC-2).^16^ In our study, prescriptions were limited to a single medication per e-prescription to assess the output regarding that particular medication.

Our dataset compiled 104 simulated scenarios with a variety of medications, routes of administration, and patient conditions, as summarized in Table 1 and Figure 2B and 2C. It comprises 99 active ingredients, 35 (33.7%) medications for continuous use, 69 (66.3%) medications for temporary use, 21 (20.2%) symptomatic medications, 12 (11.5%) antibiotics, and 6 (5.8%) psychotropics. Scenarios simulate 72 treatment indications (ICPC-2). This dataset was essential in prompting GPT-4 and Llama 3 toward a better output with instructions for the patient in individual medical prescriptions.

**Table 1:** Prompts Used to Generate Texts

Guideline	Prompt 1	Prompt 2	Prompt 3
EHR form fields to be accessed Ordered entries (EHR)	Name of patient Gender Education Disease or reason for medical prescription Medicine Dosage Total quantity dispensed	Name of patient Gender Education Disease or reason for medical prescription Medicine Dosage Total quantity dispensed	Name of patient Gender Education Disease or reason for medical prescription Medicine Dosage Total quantity dispensed
Opening command	Based on this medication [field to be inputted from dataset] and its prescribed posology [field to be inputted from dataset], generate a text instructing patients in Brazil on how to take the medication.	Based on this medication [field to be inputted from dataset] and its prescribed posology, how to use it [field to be inputted from dataset], generate a text to improve the posology of this medicine so that a patient in Brazil understands how to take it.	Based on this medication [field to be inputted from dataset] and its prescribed posology, how to use it [field to be inputted from dataset], generate a text to improve the posology of this medicine so that a patient in Brazil understands how to take it.
Additional requirement		The text must explicitly state at what time and for how long to take the medication, make recommendations on how to take it (fasting, food, water) or apply it (cleaning the area), what tools to use (spoon, hand spreading, measuring cup).	The text must explicitly state at what time and for how long to take the medication, make recommendations on how to take it (fasting, food, water) or apply it (cleaning the area), what tools to use (spoon, hand spreading, measuring cup).
Personalization requirement		Address the reader by using the person’s first name only once at the beginning. Consider the level of education field in the dataset. Use verbs in the imperative form.	Address the reader by using the person’s first name only once at the beginning. Consider the level of education field in the dataset Use verbs in imperative form.
Enhanced clarity and naturalness requirement		Generate a text as if you were a doctor, nurse, or pharmacist prescribing.	Generate a text as if you were a doctor, nurse, or pharmacist prescribing.
Enhanced clarity and naturalness requirement		The text must give clear commands to the patient, with simple sentences in the active voice. Do not use diminutive or patronizing terms.	Give clear commands to the patient, with simple sentences. Use the active voice in all sentences. Do not use diminutives or patronizing terms Do not use any sentence in the passive voice.
Enhanced Clarity and naturalness requirement			In case of continuous use medication: add “Take this medication every day. Before running out of the ones you were dispensed, make an appointment at the health unit to get a new prescription.” Instead of “to aid ingestion” use “to aid swallowing”
Acceptability requirement		Do not provide instructions on what to do if the patient forgets to take medication	Do not provide instructions on what to do if the patient forgets to take medication.
Acceptability requirement		Do not use medical jargon Do not inform about side effects or drug interactions.	Do not use medical jargon Do not inform about side effects or drug interactions. Instead of “doctor”, use “health unit.”
Acceptability requirement		Generate text only, without any preamble.	Generate text only, without any preamble.
Acceptability requirement		The text must not exceed two paragraphs	The text must not exceed two paragraphs
Bias removal requirement			Revise the text to ensure it is neutral, inclusive, and respectful, removing any implicit or explicit biases. Focus on using language that does not favor any particular gender, ethnicity, age group, culture, or ability. Replace any stereotypes with factual, unbiased descriptions, and ensure that all references to individuals or groups are made in a way that respects their dignity and diversity. Present information in a manner that is accessible and respectful to all readers, promoting equality and understanding. Avoid making assumptions, generalizations, and any language that might be considered harmful or exclusive.

EHR, electronic health record.

### Human Evaluation

Three health care professionals (Z.S.N.R., E.M.L., and C.d.S.D.) with prescriptive authority and more than 20 years of experience in medical prescribing produced a reference text for each scenario simulating a real health service using the software mockup and incorporating our set of recommendations.^15^ Upon each prompted generation, they reviewed the output texts. The interface for human analysis was implemented in the same digital mockup environment to provide a full vision of the process, as detailed in Supplemental File 3 (available online at http://www.mcpiqojournal.org).

Evaluators assessed whether the model’s generated instructions for a medication were clear and appropriate using a tool to register their observations and to confirm that the generated instructions adhered to our strict standards for clinical accuracy and patient safety. The evaluation criteria were designed to assess the adequacy, acceptability, and personalization of the instructions provided by the 3 prompts, compared with the reference text, as detailed in Supplemental File 4 (available online at http://www.mcpiqojournal.org). Furthermore, the instrument required evaluators to categorize model errors using a 7-type classification taxonomy, adapted from Roy et al,^17^ to the prescription scenario. The taxonomy and examples are available in Supplemental File 5 (available online at http://www.mcpiqojournal.org).

Upon implementing the third prompt, which required the model to remove any potential bias, a linguist assessed the output for any bias indicator.

### Outcomes and Statistics

The model’s performance was assessed using a combination of automated metrics and human validation to assess information accuracy and personalization of the output. We focused on prompt design to efficiently differentiate and rank the relative quality of models against our standard of instruction excellence according to Boubdir et al.^18^ The primary outcome of this study was a statistical analysis of the dependent comparisons among the scores from 104 evaluations conducted by physicians in 3 rounds. The secondary outcome involved using word embeddings to compare the outputs of GPT-4 and Llama 3 with reference texts, aiming to evaluate the semantic proximity between the sentences. Details are available in Supplemental File 6 (available online at http://www.mcpiqojournal.org).

## Results

Human evaluators assessed 416 paired comparisons across 3 rounds and an additional comparative round between GPT-4 and Llama 3, with 104 evaluations per prompt, as detailed in Table 2. The manual enhancements to the prompts led to progressively improved performance by GPT-4 in providing useful and comprehensible medication instructions. Prompt 3 outperformed prompt 1 (*P*=.028) and prompt 2 (*P*=.012) in terms of adequacy. Acceptability was fully or partially achieved in 94.3% of the texts generated by prompt 3. Regarding personalization, the instructions for patient use of medications in simulated primary care scenarios generated by GPT-4 received high approval rates from specialists, particularly with prompt 2 and even higher with prompt 3. Results from human evaluation show that the directions generated by GPT-4 and Llama 3 showed comparable adequacy (*P*=.818). Considering 95% CI overlap, full acceptability had comparable results between LLMs as well.

**Table 2:** Human Evaluation Scores for Outputs Generated by the Different Prompts Compared with the Reference Text

	GPT-4 Prompt 1 n (%) [95% CI]	GPT-4 Prompt 2 n (%) [95% CI]	GPT-4 Prompt 3 n (%) [95% CI]	Llama-3 Prompt 3 n (%) [95% CI]	*P* value^a^	*P* value^b^	*P* value^c^	*P* value^d^
Adequacy					.639	.028	.012	.818
Fully adequate	7 (6.7) [2.9-12.5]	4 (3.8) [1.0-7.7]	18 (17.3) [10.6-24.0]	15 (14.4) [8.6-22.0]				
Partially adequate	61 (58.7) [48.1-68.3]	69 (66.3) [57.7-75.0]	53 (51.0) [41.4-60.6]	46 (44.2) [34.9-53.8]				
Neither adequate nor inappropriate	12 (11.5) [5.8-18.3]	14 (13.5) [6.7-20.2]	9 (8.7) [3.8-14.4]	17 (16.3) [10.1-24.2]				
Partially inadequate	22 (21.2) [13.5-29.8]	14 (13.5) [7.7-20.2]	22 (21.2) [14.4-28.8]	23 (22.1) [14.9-30.7]				
Totally inadequate	2 (1.9) [0.0-4.8]	3 (2.9) [0.0-6.7]	2 (1.9) [0.0-4.8]	3 (2.9) [0.7-7.3]				
Acceptability					.003	*…*	*…*	*…*
Fully acceptable	19 (18.3) [11.5-26.0]	41 (39.4) [28.8-49.0]	61 (58.7) [49.0-67.3]	54 (51.9) [42.4-61.4]				
Partially acceptable	67 (64.4) [53.8-74.0]	58 (55.8) [46.2-66.3]	37 (35.6) [26.0-45.2]	26 (25.0) [17.4-33.9]				
Neither acceptable nor unacceptable	5 (4.8) [1.0-9.6]	1 (1.0) [0.0-2.9]	0 (0.0)	2 (1.9) [0.3-5.8]				
Partially unacceptable	13 (12.5) [6.7-19.2]	4 (3.8) [1.0-7.7]	6 (5.8) [1.9-10.6]	20 (19.20 [12.5-27.5]				
Totally unacceptable	0 (0.0)	0 (0.0)	0 (0.0)	2 (1.9) [0.3-5.8]				
Personalization					.012	<.001	<.001	*…*
Fully satisfactory	6 (5.8) [1.9-10.6]	23 (22.1) [14.4-30.8]	49 (47.1) [37.5-56.7]	23 (22.1) [14.9-30.7]				
Partially satisfactory	96 (92.3) [86.5-97.1]	80 (76.9) [68.3-84.6]	54 (51.9) [42.3-61.5]	75 (72.1) [63.0-0.81]				
Neither satisfactory nor unsatisfactory	2 (1.9) [0.0-4.8]	1(1.0) [0.0-2.9]	1(1.0) [0.0-2.9]	2 (1.9) [0.3-5.8]				
Totally unsatisfactory	0 (0.0)	0 (0.0)	0 (0.0)	4 (3.8) [1.2-8.7]				
Error type 1: instruction capable of causing people to adhere to incorrect guidance on the use of the medication	25 (24.0) [16.3-31.7]	32 (30.8) [22.1-40.4]	33 (31.7) [24.0-41.3]	32 (31.1) [22.7-40.4]	.065	.039	1.000	1.000
Error type 2: vague or incorrect conclusion	35 (33.7) [25.0-43.3]	28 (26.9) [19.2-35.6]	23 (22.1) [14.4-30.8]	12 (11.5) [6.4-18.6]	.167	.004	.302	.052
Error type 3: essential information on the use of the medication missing	46 (44.2) [33.7-53.8]	63 (60.6) [51.0-70.2]	44 (42.3) [32.7-51.9]	53 (51.0) [41.4-60.5]	.003	.874	.007	.272
Error type 4: factual, non-medical error (incorrect mathematical operations or generation in Portuguese)	1 (1.0) [0.0-3.8]	3 (2.9) [0.0-6.7]	3 (2.9) [0.0-6.7]	10 (9.6) [4.9-16.3]	.500	.500	1.000	.065
Error type 5: text unsupported by scientific evidence	6 (5.8) [1.9-10.6]	4 (3.8) [1.0-7.7]	1 (1.0) [0.0-2.9]	3 (2.9) [0.7-7.3]	.727	.125	.375	.625
Error type 6: text not responding to the requested task	94 (90.4) [84.6-95.2]	41 (39.4) [30.8-49.0]	39 (37.5) [28.8-47.1]	16 (15.4) [9.3-2.31]	<.001	<.001	.063	.001
Error type 7: incorrect information, hallucinations	2 (1.9) [0.0-4.8]	2 (1.9) [0.0-4.8]	1 (1.0) [0.0-2.9]	9 (8.7) [4.3-1.51]	1.000	1.000	1.000	.008

Ellipses indicate noncoincident categories.

a McNemar-Bowker Test, prompt 1 vs prompt 2.

b McNemar-Bowker Test, prompt 1 vs prompt 3.

c McNemar-Bowker Test, prompt 2 vs prompt 3.

d McNemar-Bowker Test, prompt 3 GPT-4 vs Llama 3.

Human evaluators identified errors in 102 (90.1%) texts generated by prompt 1, 93 (89.4%) by prompt 2, and 62 (77.9%) by prompt 3. Type I errors, which implicate adherence to incorrect guidance, occurred more frequently with specialized prompt 3 than with prompt 1 (*P*=.039; Table 2). A deeper analysis of error mitigation revealed that among the errors with prompt 3, 29 (87.9%) occurred in short-term usage prescriptions vs 4 (11.8%) in continuous use, long term prescriptions (*P*=.002). Evaluators frequently pointed out a lack of clarity for symptomatic medication usage and a failure to specify necessary pauses in hormonal prescription regimes. The most common error, type 6, where the LLM failed to respond adequately to the task, occurred in 90.4% of texts generated by prompt 1. GPT-4 showed progressively better performance with more detailed commands in prompt 2 and prompt 3 (*P*<.001 for both). Errors such as types 4, 5, and 7 were infrequent and unaffected by our prompt design. Examples of these errors are detailed in Supplementary File 3, and samples of error-free LLM output are in provided Supplementary File 4. Comparing the LLMs output in prompt 3, we found no significant difference regarding error types 1, 2, 3, or 4. Texts generated by Llama 3 were better adjusted to the requested tasks than those generated by GPT-4 because they had fewer instances of error type 6 (*P*=.001). However, hallucinations or incorrect information (error type 7) was more frequent in texts generated by Llama 3 than in those generated by GPT-4 (8.7% vs 1.0%, *P*=.008, respectively).

For GPT-4, gender bias was found in 6 of the 104 texts generated with prompt 1, in all 104 texts generated with prompt 2, and none of the texts generated with prompt 3. Bias was found in using the masculine grammatical gender in words in Portuguese to refer to the generic entities “physician” and “pharmacist.” For Llama 3, no gender bias was found. This points to the positive impact of explicit bias removal in the prompt design, as carried out in prompt 3.

Objective automated comparisons demonstrated significant differences in similarity rates between the reference texts and GPT-4 outputs generated by the different prompts (*P*=.001), as shown in Table 3. Pairwise comparisons of metrics indicated that the similarity rate of prompt 1 was lower than those of prompts 2 and 3. However, there was no significant difference between the mean similarity rates of prompts 2 and 3. Llama 3 and GPT-4 output generated by prompt 3 showed no difference in terms of similarity when compared with the reference text.

**Table 3:** Similarity Rate Comparisons Between Reference Text and Large Language Model Outputs Generated by Different Prompts

	Mean (SD)	Comparisons	Difference [95% CI]	*P* value^a^
Prompt 1	80.6 (4.7)	Prompt 2	−10.4 [−11.8 to −9.0]	<.001
		Prompt 3	−10.8 [−12.2 to −9.4]	<.001
Prompt 2	91.0 (4.1)	Prompt 1	10.4 [9.0 to 11.8]	<.001
		Prompt 3	−0.39 [−1.8 to 1.0]	.517
Prompt 3	91.4 (3.8)	Prompt 1	10.8 [9.4 to 12.2]	<.001
		Prompt 2	0.39 [−1.0 to 1.8]	.517
	91.4 (3.7)	Llama-3, prompt 3	0.356 [−0.18 to 0.18]	0.703^b^

Repeated measures analysis of variance, *P*<.001; Mauchly’s W, 0.617; sphericity assumed.

a Post hoc Bonferroni test.

b T-mean test.

However, cases of high similarity rates do not implicate correct directions. For instance, directions for spiramycin prescribed to a pregnant woman achieved a similarity rate of 94.75% with prompt 1 (Table 3) despite being partially inadequate and containing error types 5, 6, and 7 (hallucinations) as identified by specialists (Table 2).

## Discussion

Investments in e-prescribing systems are strongly supported by scientific evidence.^4^ Recognized benefits include saving time for physicians, pharmacists, and patients; reducing risks of errors due to illegible prescriptions; simplifying processes for repeat prescriptions in chronic diseases; improving health care quality; standardizing language; ensuring inclusion of essential pharmacotherapeutic information; and reducing costs for national health systems.^6^^,^^19^^,^^20^ Intelligent prescription systems harness the potential of cognitive behavioral approaches to meet individual patient needs. Such tasks demand communication skills, language simplification, and personalization, which are time-intensive for already overwhelmed health professionals.^21^

The main contribution of our study lies in pointing to the potential use of LLMs to generate texts to support prescribers and patients, leading to more effective communication between front-line primary care health professionals and people in their assigned territories. The initial step involved defining the requirements for adequacy, acceptability, and personalization of tailored instructions for medication use. The outcome was an online accessible set of guidelines in Portuguese refined through successive rounds of evaluation.^15^ These guidelines were designed to generate specific instructions in a variety of scenarios encompassing 99 active ingredients, different frequencies of use, different preparations, 14 administration routes, and 77 treatment indications using ICPC-2.^16^ It should be acknowledged, however, that there is a very large number of active ingredients, pharmaceutical forms, routes, and treatment indications in primary health care and that this diversity will ultimately be targeted in studies involving real scenarios. For the time being, we have used a simulated dataset developed by specialists and pursued evolving prompts to output texts that are adequate, acceptable, and personalized while bearing in mind our reference texts. Our results corroborate the emergent paradigm that LLMs are transforming our understanding of natural language with applications in digital health.^22^ Designing appropriate prompts is crucial for enhancing model performance on downstream tasks because a well-designed prompt provides clear guidance and facilitates effective task completion.^22^

Even in a pilot version with a simulated dataset, our mockup software can be expanded with more scenarios, a diverse range of prescriptions, and input from a larger team of specialists to achieve optimal performance. In addition to providing a proper prescription, prescribers are expected to explain how to use medications. However, personalization for safe medication use has not yet reached a consensus among governments and pharmacy guidelines. This project proposed a standard based on literature and local practices, including warnings against the culture of patients sharing medications with relatives and friends. Nevertheless, as a first step toward a comprehensive model, the results, although requiring further improvement, highlight the potential of LLMs to enhance the communication between health professionals and patients verbally mediated and documented by EHR prescription. This is relevant because in clinical practice, directions in prescriptions are not always complete and fully comprehensible by the patient.^7^ Nonetheless, generated directions are not meant to replace actual medical advice.

We suggest caution when interpreting our results because our dataset, although realistic, is based solely on our experiences with prescriptions and needs to be expanded to encompass a wider range of experiences including other datasets and prescribers. Further improvement can be achieved by incorporating additional data from the national e-SUS Primary Care system such as race, gender identity, occupation, and city of residence to enhance personalization. Interrater agreement and reproducibility of LLM generated texts were not targeted in our study. Incremental solutions in the reference directions for medication use are still necessary to achieve broad generalizability. Prompting LLMs to produce unbiased texts requires a diverse range of patients, including various races, gender identities, and minority groups of traditional peoples, such as indigenous peoples, quilombola communities, artisanal fishermen, gypsy peoples, and others. Furthermore, developed prompts require enhancements to face challenges as the following:

• Addressing the need for medication prescriptions upon symptom occurrence, such as fever, pain, or nausea, introducing these data as input in the model.
• Providing specific instructions on medications that require dosage adjustments over time.
• Indicating specific instructions for sequential tablets used in medication regimes, such as oral contraceptives.
• Customizing usage directions for medications prescribed for either continuous or limited-time use, tailored to individual patient needs, such as aspirin or rivaroxaban.
• Developing solutions for issues arising from a lack of fields in standard EHR data inputs, such as a field to specify body parts for topical medications.
• Exploring LLM-based solutions applied to mobile applications and enhancing accessibility and convenience for health care providers on the move.

Prompt design for tasks involving natural language processing has demonstrated significant benefits for the health care sector, offering more efficient and cost-effective ways to use LLMs for specific tasks.^22^ In our study, human evaluation played a crucial role in the iterative improvement of prompts 1, 2, and 3, as well as comparisons between 2 LLMs. The comparison between open-source LLMs (Llama 3) and closed-source ones (GPT-4) is academically relevant because the latter are black boxes regarding how they were trained or what their internal structure is like. Moreover, closed-source LLMs raise issues regarding sensitive data (ie, patients’ information), which may violate privacy issues.^23^ Our study showed that open-source LLMs results can be as good as closed-source ones.

There is still potential to improve prompting to enhance acceptability and reduce errors. Moreover, the continuous evolution of LLMs necessitates ongoing adjustments to better serve the health care domain, particularly in languages such as Portuguese. One recurrent issue identified by specialists is translation from English to Portuguese, which affected the naturalness of the output language. For example, passive voice sentences of the type “you have been prescribed” are not natural in Portuguese.

Complementary approaches are being developed to address these issues. Prompt tuning, an innovative method that operates within the embedding space rather than being limited to text-readable forms, offers a promising automated solution. This technique allows for the optimization and fine-tuning of training data, enhancing the efficiency and accuracy of language models in executing medical tasks, which is in agreement with the previous report.^22^ However, to overcome the constraints of our current prompt system, implementing retrieval-augmented generation is still a critical goal, notably for its capability for streamlined knowledge acquisition. By adopting this approach, we aim to significantly enhance our prompts by linking them to dependable external knowledge sources.^23^ This will not only enrich the instructions for specific treatments, such as hormones and contraceptives, but also foster more accurate and contextually appropriate content. Additionally, retrieval-augmented generation is expected to improve the responses concerning the particularities of medications in specialized fields such as ophthalmology, dermatology, and otolaryngology. It will also better accommodate unusual routes of administration, such as transdermal applications and suspensions, ensuring that the generated instruction is both easy to understand and contextually appropriate.

The potential impact of enhanced and effective communication between health professionals and patients on the quality of care could be substantial. Brazil’s public health care system (e-SUS) serves more than 190 million people, 80% of whom rely exclusively on these public services for their health care needs, which are guaranteed free of charge by the Federal Constitution.^24^ Most primary care units have transitioned from traditional to electronic prescribing through the e-SUS Primary Care system. Our efforts were specifically directed toward a future AI implementation in the national e-prescribing systems within the EHR framework. Furthermore, according to the Statistical Yearbook of the Pharmaceutical Market, in 2022, the Brazilian market saw the sale of 5,710,898,201 packages of 4748 different products, representing a huge opportunity to introduce improvements with appropriate AI technologies.^25^ Artificial intelligence in health care can automate tasks, improve communication, reduce cultural biases, and promote patient health management, thereby serving as a complementary tool that supports rather than replaces the expertise and judgment of physicians.^26^

Our initiative aligns with ethical principles and safety guidelines for AI implementation in health care,^27^ moving beyond traditional textual simplification to develop an intelligent, multimodal response system that incorporates both written and spoken formats using natural language. By enhancing the clarity and safety of medication communication, dosage instructions, and usage methodologies effectively communicated, we have contributed to patient health literacy while preserving the autonomy of health care professionals to adopt and adapt our solution. In the future, we expect narrative electronic prescribing instructions to advance health literacy, as reported already in other studies.^28^ Moreover, other Portuguese-speaking countries could benefit from the proposed language models. Besides, the potential for LLM-based solutions extends to mobile applications, enhancing accessibility and convenience for health care providers on the move.

## Conclusion

This study demonstrates the potential of LLM-supported generation to produce prescription directions and improve communication between health professionals and patients within the e-prescribing system.

## Potential Competing Interests

The authors report no competing interests.
