# Supplementary material for: Evaluating Large Language Model–Supported Instructions for Medication Use: First Steps Toward a Comprehensive Model
Source: Mayo Clin Proc Digit Health. 2024 Oct 19;2(4):632–44. doi: 10.1016/j.mcpdig.2024.09.006 (PMC11638470; doi:10.1016/j.mcpdig.2024.09.006)
Supplement: Supplementary Data [file mmc1.pdf]

														Prompt1				Prompt1_ERROR							Prompt2				Prompt2_ERROR							GPT4-Prompt3				GPT4-Prompt3_error							Llama3-Prompt3				Llma3-Prompt3-Error						
N	Name	Cat_AGE	Age-Years	Sex	Education	Demand	Psicotropicos	Preparation	Route_corr	Route	Prescrição	Use_Term	Symptom_drug	Similarity	Adequacy	Aceptability	Personalization	1	2	3	4	5	6	7	Similarity	Adequacy	Aceptability	Personalization	1	2	3	4	5	6	7	Similarity	Adequacy	Aceptability	Personalization	1	2	3	4	5	6	7	Similarity	Adequacy	Aceptability	Personalization	1	2	3	4	5	6	7
1	Ana Beatriz da Silva Ribeiro Cardoso Ferreira	2.00	25	female	High School	INSUFICIÊNCIA CARDÍACA - K77	0.00	Pills	10.00	Oral	Medicamentos<br>Ácido Acetilsalicílico 100 mg Pills Pills<br>180 PillsS DE LIBERAÇÃO PROLONGADA<br>Posologia:<br>1 Pills. 1 vez ao dia. período indeterminado	LT_medication	0	79.37	2	2	2	0	0	1	0	0	1	0	90.94	2	1	1	0	0	1	0	0	0	0	93.49	2	1	1	0	0	0	0	0	0	0	91.78	4.00	1.00	2.00	0.00	0.00	0.00	0.00	1.00	0.00	0.00
2	Ana Clara da Silva Santos Ferreira Fujimoto	3.00	82	female	Complete Primary Education	GRAVIDEZ - W78	0.00	Pills	10.00	Oral	Medicamentos<br>Ácido Fólico 2 mg Pills Pills<br>90 PillsS<br>Posologia:<br>1 Pills. 1 vez ao dia. por 3 meses	ST_medication	0	86.90	2	2	2	0	0	1	0	0	1	0	98.58	1	2	1	0	0	0	0	0	0	0	94.12	2	1	1	0	0	1	0	0	0	0	94.18	3.00	2.00	2.00	0.00	1.00	0.00	0.00	0.00	1.00	0.00
3	Ana Clara Ferreira Santos Mendes Choi	2.00	25	female	University. Diploma. Master's. Doctoral degree	ICTERÍCIA - D13	0.00	Pills	10.00	Oral	Medicamentos<br>Ácido Ursodesoxicólico 150 mg Pills Pills<br>90 PillsS<br>Posologia:<br>3 Pillss. 1 vez ao dia. período indeterminado	LT_medication	0	84.47	2	2	2	0	1	0	0	0	1	0	95.88	2	2	2	0	0	0	0	0	0	0	94.41	2	2	1	0	0	1	0	0	0	0	93.05	2.00	1.00	2.00	0.00	0.00	1.00	0.00	0.00	0.00	0.00
4	Ana Clara dos Santos Silva Pereira Matos	2.00	37	female	Adult literacy course	OSTEOPOROSE - L95	0.00	Pills	10.00	Oral	Medicamentos<br>Alendronato de Sódio + Carbonato de Cálcio + Colecalciferol 70 mg + 500 mg + 200 ui Caixa Pills<br>8 PillsS<br>Posologia:<br>1 Pills. 1 vez a cada 15 dias. período indeterminado	LT_medication	0	67.87	2	1	2	0	0	1	0	0	0	0	72.67	3	2	2	1	0	1	0	1	0	0	78.22	2	1	1	0	0	1	0	0	0	0	94.17	2.00	1.00	1.00	0.00	0.00	0.00	0.00	0.00	0.00	0.00
5	Tiago Oliveira Santos Pereira Lima	2.00	51	male	Elementary Education 4th to 8th grades	DISTÚRBIO ANSIOSO/ESTADO DE ANSIEDADE - P74	1.00	Pills	10.00	Oral	Medicamentos<br>Alprazolam 0.25 mg Pills Pills<br>30 PillsS<br>Posologia:<br>1 Pills. 3 vezes ao dia. período indeterminado	LT_medication	0	84.96	2	2	2	0	0	1	0	0	0	0	89.86	2	1	2	0	0	1	0	0	0	0	92.76	2	1	2	0	0	1	0	0	0	0	91.67	4.00	1.00	3.00	1.00	1.00	1.00	1.00	0.00	0.00	0.00
6	Lívia Silva Fonseca Pires Nobre	2.00	46	female	No schooling	DERMATOFITOSE - S74	0.00	Esmalte	3.00	Dermatologica	Medicamentos<br>Amorolfina. Cloridrato 50 mg/ml Frasco 2.5 ml Esmalte<br>2 FRASCOS<br>Posologia:<br>1 aplicação. 1 vez a cada 1 semana. durante 6 meses	ST_medication	0	81.01	2	4	2	1	0	0	0	0	1	0	90.59	2	2	2	1	0	0	0	0	1	0	89.92	2	2	2	1	0	1	0	0	1	0	91.32	2.00	1.00	2.00	0.00	0.00	0.00	0.00	0.00	0.00	0.00
7	Ana Luiza Souza Menezes Oliveira Silva	2.00	50	female	High School	CISTITE/OUTRA INFECÇÃO URINÁRIA - U71	0.00	Pills	10.00	Oral	Medicamentos<br>Amoxicilina + Clavulanato de Potássio 875 mg + 125 mg Pills Pills<br>14 PillsS<br>Posologia:<br>1 Pills. a cada 12 horas. durante 7 dias	ST_medication	0	80.60	2	2	1	0	0	1	0	0	1	0	88.36	2	1	2	0	0	1	0	0	0	0	89.59	2	1	1	0	0	0	0	0	0	0	92.59	1.00	1.00	2.00	0.00	0.00	0.00	0.00	0.00	0.00	0.00
8	Leonardo Santos Silva Moraes Fujimoto	2.00	39	male	Elementary Education 4th to 8th grades	AMIGDALITE AGUDA - R76	0.00	capsule	10.00	Oral	Medicamentos<br>Amoxicilina 500 mg Cápsula Cápsula<br>30 CÁPSULAS<br>Posologia:<br>1 cápsula. a cada 8 horas. durante 10 dias	ST_medication	0	78.11	3	2	2	1	1	0	0	0	1	0	90.59	2	2	2	1	1	0	0	0	1	0	92.73	2	2	1	1	1	0	0	0	1	0	94.13	2.00	1.00	2.00	0.00	0.00	0.00	0.00	0.00	0.00	0.00
9	Ricardo Santos de Oliveira Silva	1.00	16	male	No schooling	HIPERTENSÃO SEM COMPLICAÇÕES - K86	0.00	Pills	10.00	Oral	Medicamentos<br>Anlodipino. Besilato 5 mg Pills Pills<br>90 PillsS<br>Posologia:<br>1 Pills. 1 vez ao dia. periodo indeterminado	LT_medication	0	83.64	2	2	2	0	1	1	0	0	1	0	96.87	2	2	1	1	1	1	0	0	0	0	90.62	2	2	1	0	0	1	0	0	0	0	78.54	4.00	1.00	2.00	1.00	0.00	0.00	1.00	0.00	0.00	0.00
10	Thiago Santos Oliveira da Silva	2.00	32	male	Elementary Education 1st to 4th grades	ALTERAÇÃO NO METABOLISMO DOS LÍPIDOS - T93	0.00	Pills	10.00	Oral	Medicamentos<br>Atorvastatina Cálcica 20 mg Pills Pills<br>90 PillsS<br>Posologia:<br>1 Pills. 1 vez ao dia. periodo indeterminado	LT_medication	0	82.15	3	2	2	0	0	0	0	0	1	0	92.89	4	1	2	0	0	0	0	0	1	0	94.29	4	1	2	0	0	0	0	0	1	0	94.59	4.00	4.00	2.00	0.00	0.00	1.00	0.00	0.00	0.00	0.00
11	Luciana Santos Silva Matos Kimura	2.00	50	female	High School	SINUSITE CRÔNICA/AGUDA - R75	0.00	Pills	10.00	Oral	Medicamentos<br>Azitromicina 500 mg Pills Pills<br>6 PillsS<br>Posologia:<br>1 Pills. a cada 12 horas. durante 3 dias	ST_medication	0	74.95	2	4	2	0	1	0	0	0	1	0	92.44	2	4	2	0	0	0	0	0	1	0	93.72	2	1	2	1	1	0	0	0	1	0	93.54	2.00	1.00	2.00	0.00	0.00	0.00	0.00	0.00	0.00	0.00
12	Eduardo Silva de Almeida Ferreira Santos	2.00	43	male	Adult literacy course	DOENÇA CARDÍACA ISQUÊMICA COM ANGINA - K74	0.00	Pills	10.00	Oral	Medicamentos<br>Bisoprolol. Fumarato 5 mg Pills Pills<br>30 PillsS<br>Posologia:<br>1 Pills. pela manhã. uso contínuo	LT_medication	0	77.59	3	4	2	0	0	0	0	0	1	0	93.92	2	1	2	1	0	0	0	0	1	0	92.81	4	1	2	0	0	0	0	0	1	0	90.50	2.00	2.00	2.00	0.00	0.00	1.00	0.00	0.00	0.00	0.00
13	João Paulo Silva Oliveira Santos	2.00	54	male	High School	ABUSO DO TABACO - P17	1.00	Pills	10.00	Oral	Medicamentos<br>Bupropiona. Cloridrato 150 mg Pills Pills<br>180 PillsS<br>Posologia:<br>1 Pills. pela manhã. período indeterminado	LT_medication	0	86.57	2	2	2	0	0	1	0	0	1	0	92.31	2	2	2	0	0	1	0	0	0	0	92.47	1	1	1	0	0	0	0	0	0	0	89.05	4.00	4.00	2.00	0.00	0.00	1.00	0.00	0.00	0.00	0.00
14	Lucas Almeida Silva Souza Santos	2.00	43	male	High School	INFECÇÃO PÓS-TRAUMÁTICA DA PELE - S11	0.00	capsule	10.00	Oral	Medicamentos<br>Cefalexina 500 mg Cápsula Cápsula<br>28 CÁPSULAS<br>Posologia:<br>1 cápsula. a cada 6 horas. durante 7 dias	ST_medication	0	80.39	2	2	2	0	0	1	0	0	1	0	94.95	4	2	2	1	1	1	0	0	0	0	91.39	3	1	1	1	0	0	0	0	0	0	94.91	2.00	1.00	1.00	0.00	0.00	0.00	0.00	0.00	0.00	0.00
15	Letícia Melo Santos de Oliveira	2.00	44	female	Elementary Education 4th to 8th grades	DORES MENSTRUAIS - X02	0.00	capsule	10.00	Oral	Medicamentos<br>Celecoxibe 200 mg Cápsula Cápsula<br>8 CÁPSULAS<br>Posologia:<br>1 cápsula. 2 vezes ao dia. durante 3 dias	ST_medication	1	83.61	4	4	2	1	0	1	0	0	1	0	90.29	3	1	2	1	0	1	0	0	1	0	90.41	4	4	2	1	0	1	0	0	1	0	88.01	2.00	2.00	2.00	1.00	0.00	1.00	0.00	0.00	0.00	0.00
16	Ana Maria Ferreira de Carvalho Li da Silva	2.00	25	female	High School	DORES MUSCULARES - L18	0.00	Pills	10.00	Oral	Medicamentos<br>Ciclobenzaprina. Cloridrato 10 mg Pills Pills<br>5 PillsS<br>Posologia:<br>1 Pills. pela noite. durante 5 dias	ST_medication	0	80.22	2	1	2	0	0	0	0	0	1	0	90.64	3	2	2	1	0	0	0	0	1	0	95.02	2	1	1	0	0	0	0	0	1	0	87.11	2.00	4.00	2.00	0.00	0.00	1.00	0.00	0.00	0.00	0.00

17	Letícia Almeida de Oliveira Santos	2.00	48	female	High School	DERMATITE/ECZEMA ATÓPICO - S87	0.00	Pomada	3.00	Dermatologica	Medicamentos<br>Betametasona. Dipropionato 0.5 mg/g Bisnaga 30 g Creme<br>1 BISNAGAS<br>Posologia:<br>1 aplicação. 2 vezes ao dia. durante 4 semanas	ST_medication	0	81.75	1	2	2	0	0	0	0	0	1	0	86.01	2	2	2	0	0	1	0	0	1	0	85.34	1	2	2	0	0	0	0	0	1	0	90.98	2.00	2.00	2.00	0.00	0.00	0.00	0.00	0.00	0.00	0.00
18	Carla Santos da Silva Siqueira Fujimoto	2.00	20	female	Adult literacy course	ASMA - R96	0.00	Aerosol	1.00	Aerosol nasal	Medicamentos<br>Budesonida 100 mcg/dose Frasco 100 doses Pó para inalação oral<br>1 FRASCOS<br>Posologia:<br>2 aplicações. 2 vezes ao dia. período indeterminado	LT_medication	0	81.09	2	2	2	0	0	0	0	0	1	0	91.02	2	2	2	0	0	1	0	0	0	0	90.93	2	1	1	0	0	1	0	0	0	0	86.70	4.00	2.00	1.00	1.00	0.00	1.00	0.00	0.00	0.00	1.00
19	Juliana Gomes da Silva Moreira Santos	2.00	29	female	University. Diploma. Master's. Doctoral degree	SENSAÇÕES OCULARES ANORMAIS - F13	0.00	Solução o	9.00	Oftalmica	Medicamentos<br>Carmelose Sódica 5 mg/ml Frasco 5 ml Solução oftálmica<br>1 FRASCOS<br>Posologia:<br>1 gota. 1 vez ao dia. período indeterminado	LT_medication	1	83.58	4	1	2	0	1	0	0	0	1	0	91.19	3	2	2	1	0	0	0	0	1	0	91.08	2	2	2	1	0	1	0	0	1	0	88.26	4.00	4.00	2.00	0.00	0.00	1.00	0.00	0.00	0.00	0.00
20	Leonardo Silva Andrade Pereira da Costa	2.00	33	male	Complete Primary Education	DERMATITE SEBORREICA - S86	0.00	Xampu	2.00	Capilar	Medicamentos<br>Cetoconazol 2 % Frasco 120 ml Xampu<br>1 FRASCOS<br><br>Posologia:<br>1 aplicação. 2 vezes por semana. por 1 mês	ST_medication	0	74.05	2	2	2	0	0	1	0	0	1	0	94.20	2	1	2	0	0	0	0	0	0	0	90.12	2	1	1	0	0	0	0	0	0	0	95.28	3.00	1.00	2.00	0.00	0.00	1.00	0.00	0.00	0.00	0.00
21	João Carlos Miramontes Silva Neto	2.00	27	male	University. Diploma. Master's. Doctoral degree	CEFALÉIA - N01	0.00	Ampola	7.00	Intramuscular	Medicamentos<br>Cetoprofeno 50 mg/ml Ampola 2 ml Solução injetável<br>2 AMPOLAS<br><br>Posologia:<br>2 ampolas. dose única. durante 1 dia	ST_medication	1	79.17	4	2	2	1	1	0	0	0	1	0	76.97	5	2	2	1	1	1	0	1	1	0	86.05	2	2	2	1	0	1	0	0	1	0	89.99	2.00	1.00	2.00	0.00	0.00	0.00	0.00	0.00	0.00	0.00
22	Carlos Alberto Silva Souza Pereira Lima	2.00	41	male	Complete Primary Education	DERMATOFITOSE - S74	0.00	Solução t	3.00	Dermatologica	Medicamentos<br>Ciclopirox Olamina 10 mg/ml Frasco 15 ml Solução tópica<br>2 ML<br><br>Posologia:<br>4 gotas. 2 vezes ao dia. duante 30 dias	ST_medication	0	81.45	5	2	2	1	1	1	0	0	1	1	86.53	5	2	3	1	1	1	1	0	0	1	86.61	5	2	2	1	1	0	1	0	0	1	95.65	1.00	1.00	1.00	0.00	0.00	0.00	0.00	0.00	0.00	0.00
23	Felipe dos Santos Pereira Nogueira da Silva	2.00	48	male	Elementary Education 4th to 8th grades	CONJUNTIVITE INFECCIOSA - F70	0.00	Solução o	9.00	Oftalmica	Medicamentos<br>Ciprofloxacino + Dexametasona 3.5 + 1 mg/ml Frasco 5 ml Solução oftálmica<br>1 FRASCOS<br><br>Posologia:<br>1 gota. a cada 4 horas. durante 7 dias	ST_medication	0	83.26	3	1	2	1	0	0	0	0	1	0	88.96	2	1	2	1	0	1	0	0	1	0	88.72	2	1	2	1	0	1	0	0	1	0	92.75	4.00	4.00	2.00	0.00	0.00	1.00	0.00	0.00	0.00	0.00
24	Roberto Silva Santos Pereira Chen	1.00	15	male	No schooling	OTITE EXTERNA - H70	0.00	Suspensã	11.00	Otologica	Medicamentos<br>Ciprofloxacino + Hidrocortisona 2 + 10 mg/ml Frasco 5 ml Suspensão otológica<br>1 FRASCOS<br><br>Posologia:<br>3 gotas. a cada 12 horas. durante 7 dias	ST_medication	0	88.01	2	2	2	1	1	0	0	0	1	0	93.20	2	2	2	1	1	0	0	0	1	0	91.11	2	1	2	1	1	0	0	0	0	0	92.86	2.00	2.00	2.00	0.00	0.00	0.00	0.00	0.00	1.00	0.00
25	Lívia Mendes Ferreira Souza Silva	2.00	20	female	Complete Primary Education	QUEDA DE CABELO/CALVÍCIE - S23	0.00	capsule	10.00	Oral	Medicamentos<br>Cistina + Ácido Aminobenzóico + Pantotenato de Cálcio + Nitrato de Tiamina + Levedura + Queratina 60 + 20 + 60 + 100 + 20 + 20 mg Cápsula Cápsula<br>180 CÁPSULAS<br><br>Posologia:<br>1 cápsula. 2 vezes ao dia. período indeterminado	LT_medication	0	68.16	2	2	2	0	1	1	0	0	1	0	91.87	2	2	2	0	0	1	0	0	1	0	92.58	2	1	2	0	0	0	0	0	1	0	94.69	1.00	4.00	2.00	0.00	0.00	1.00	0.00	0.00	0.00	0.00
26	João Carlos Souza Silva Kimura	2.00	38	male	High School	INFECÇÃO ESTREPTOCÓCICA DA OROFARINGE - R72	0.00	capsule	10.00	Oral	Medicamentos<br>Claritromicina 500 mg Pills Pills<br>14 CÁPSULAS<br><br>Posologia:<br>1 cápsula. a cada 12 horas. durante 7 dias	ST_medication	0	82.03	2	2	2	0	0	0	0	0	1	0	94.84	2	2	2	0	0	1	0	0	0	0	93.51	1	1	1	0	0	0	0	0	0	0	92.80	2.00	1.00	2.00	0.00	0.00	0.00	0.00	0.00	0.00	0.00
27	Gabriela de Oliveira Nascimento Silva	3.00	66	female	University. Diploma. Master's. Doctoral degree	SINUSITE CRÔNICA/AGUDA - R75	0.00	capsule	10.00	Oral	Medicamentos<br>Clindamicina. Cloridrato 300 mg Cápsula Cápsula<br>20 CÁPSULAS<br><br>Posologia:<br>1 cápsula. a cada 12 horas. durante 10 dias	ST_medication	0	81.26	2	2	2	0	0	0	0	0	1	0	89.97	2	1	2	0	0	1	0	0	0	0	95.00	2	1	1	0	0	1	0	0	0	0	83.67	3.00	2.00	2.00	1.00	1.00	1.00	0.00	0.00	0.00	1.00
28	Eduardo Silva Santos Moura Lima	2.00	31	male	High School	DERMATITE DE CONTATO/ALÉRGICA - S88	0.00	Creme	3.00	Dermatologica	Medicamentos<br>Clobetasol. Propionato 0.5 mg/g Bisnaga 30 g Creme<br>1 BISNAGAS<br><br>Posologia:<br>1 aplicação. 2 vezes ao dia. durante 7 dias	ST_medication	0	81.25	4	1	2	0	0	0	0	0	1	0	85.44	2	1	2	0	0	0	0	0	1	0	83.83	4	4	2	0	0	1	0	0	1	0	88.62	2.00	3.00	2.00	0.00	0.00	1.00	0.00	0.00	0.00	0.00

29	Lucas Oliveira Santos da Silva	3.00	61	male	University. Diploma. Master's. Doctoral degree	DEFICIÊNCIA VITAMÍNICA/NUTRICIONAL - T91	0.00	capsule	10.00	Oral	Medicamentos<br>Colecalciferol (Vitamina D3) 7.000 ui Cápsula Cápsula<br>30 CÁPSULAS<br><br>Posologia:<br>1 cápsula. 1 vez a cada 7 dias. período indeterminado	LT_medication	0	77.45	4	4	2	0	0	0	0	0	1	0	92.52	2	1	2	0	0	0	0	0	1	0	93.84	4	1	2	0	0	0	0	0	1	0	93.87	2.00	1.00	2.00	0.00	0.00	1.00	0.00	0.00	0.00	0.00
30	Leonardo Rocha da Silva Nascimento Almeida	2.00	33	male	High School	DIABETES 2 INSULINO-DEPENDENTE - T90	0.00	Pills	10.00	Oral	Medicamentos<br>Dapagliflozina 10 mg Pills Pills de liberação controlada<br>90 PillsS<br><br>Posologia:<br>1 Pills. pela manhã. período indeterminado	LT_medication	0	71.48	2	2	2	0	1	1	0	0	1	0	89.20	2	1	2	0	0	1	0	0	0	0	96.49	1	1	1	0	0	0	0	0	0	0	94.12	2.00	1.00	1.00	0.00	0.00	0.00	0.00	0.00	1.00	0.00
31	Rodrigo de Oliveira Santos Silva	3.00	61	male	Elementary Education 4th to 8th grades	PSORÍASE - S91	0.00	Pills	10.00	Oral	Medicamentos<br>Deflazacorte 30 mg Pills Pills<br>10 PillsS<br><br>Posologia:<br>1 Pills. 2 vezes ao dia. durante 5 dias	ST_medication	0	81.10	4	1	2	0	0	0	0	0	1	0	88.50	2	1	2	0	0	0	0	0	1	0	86.23	4	1	2	0	0	0	0	0	1	0	94.81	2.00	1.00	1.00	0.00	1.00	0.00	1.00	0.00	0.00	1.00
32	Leonardo Silva Melo Castro Souza	2.00	54	male	High School	RINITE ALÉRGICA - R97	0.00	Pills	10.00	Oral	Medicamentos<br>Desloratadina 5 mg Pills Pills<br>7 PillsS<br><br>Posologia:<br>1 Pills. 1 vez ao dia. durante 1 semana	ST_medication	0	69.07	2	2	2	0	0	1	0	0	0	0	89.45	2	2	2	0	1	0	0	0	0	0	92.58	1	2	1	0	0	0	0	0	1	0	95.29	1.00	2.00	2.00	0.00	0.00	0.00	0.00	0.00	0.00	0.00
33	Lucas Silva Pereira Mendes Oliveira	3.00	61	male	High School	BLEFARITE/HORDÉOLO/CALÁZIO - F72	0.00	Solução o	9.00	Oftalmica	Medicamentos<br>Dexametasona 1 mg/ml Frasco 5 ml Solução oftálmica<br>1 ML<br><br>Posologia:<br>1 gota. a cada 4 horas. durante 3 semanas	ST_medication	0	82.80	2	2	2	0	1	0	0	0	1	0	93.26	2	2	2	0	1	1	0	0	0	0	95.05	2	2	2	0	1	0	0	1	0	0	91.75	4.00	1.00	5.00	0.00	0.00	1.00	0.00	0.00	0.00	0.00
34	Rafael Gomes Almeida Moura Santos	3.00	71	male	High School	ALERGIA/REAÇÃO ALÉRGICA NE - A92	0.00	Pills	10.00	Oral	Medicamentos<br>Dexclorfeniramina. Maleato 2 mg Pills Pills<br>28 PillsS<br><br>Posologia:<br>1 Pills. a cada 6 horas. durante 7 dias	ST_medication	0	75.79	3	2	2	0	1	1	0	0	1	0	89.78	2	2	2	0	0	1	0	0	1	0	92.62	4	1	2	0	1	1	0	0	0	0	88.72	1.00	4.00	2.00	0.00	0.00	1.00	0.00	0.00	0.00	0.00
35	Mariana Silva Santos Nogueira Yang	2.00	24	female	Elementary Education 4th to 8th grades	DISTÚRBIO ANSIOSO/ESTADO DE ANSIEDADE - P74	1.00	Pills	10.00	Oral	Medicamentos<br>Diazepam 5 mg Pills Pills<br>30 PillsS<br><br>Posologia:<br>1 Pills. pela noite. durante 60 dias	ST_medication	0	81.86	3	4	2	1	0	1	0	0	1	0	80.63	2	4	2	1	0	1	0	0	1	0	83.11	3	4	2	1	0	1	0	0	1	0	85.98	5.00	3.00	2.00	1.00	1.00	0.00	0.00	0.00	1.00	1.00
36	Carlos Eduardo Pereira da Silva Gonzaga Andrade	2.00	34	male	High School	TORÇÕES E DISTENSÕES DO TORNOZELO	0.00	Pills	10.00	Oral	Medicamentos<br>Diclofenaco Potássico 50 mg Pills Pills<br>9 PillsS<br><br>Posologia:<br>1 Pills. 3 vezes ao dia. durante 3 dias	ST_medication	1	82.50	4	1	2	0	0	0	0	0	1	0	85.70	4	4	2	0	0	0	0	0	1	0	88.03	4	4	2	0	0	0	0	0	1	0	93.09	2.00	1.00	2.00	0.00	0.00	0.00	0.00	0.00	1.00	0.00
37	Alessandra Santos da Silva Pereira	2.00	20	female	High School	DERMATOFITOSE - S74	0.00	Creme	3.00	Dermatologica	Medicamentos<br>Clotrimazol + Dexametasona 10 mg + 0.4 mg/g Bisnaga 40 g Creme<br>1 BISNAGAS<br><br>Posologia:<br>1 bisnaga. 2 vezes ao dia. durante 2 semanas	ST_medication	0	84.36	2	2	2	0	1	0	0	0	1	0	93.25	1	2	1	0	0	0	0	0	0	0	94.31	2	2	1	0	0	1	0	0	0	0	88.17	2.00	1.00	2.00	0.00	0.00	0.00	0.00	0.00	0.00	0.00
38	Lucas da Silva Ribeiro Lee Gonçalves	1.00	18	male	Elementary Education 4th to 8th grades	GRIPE - R80	0.00	Pills	10.00	Oral	Medicamentos<br>Dipirona Sódica 1 g Pills Pills<br>20 PillsS<br><br>Posologia:<br>1 Pills. a cada 6 horas. duratne 5 dias	ST_medication	1	79.84	3	2	2	1	0	1	0	0	0	0	80.06	3	2	2	0	0	1	0	0	0	0	74.55	2	1	1	1	0	0	0	0	0	0	92.46	2.00	1.00	2.00	0.00	0.00	0.00	0.00	0.00	0.00	0.00
39	Leonardo Silva Ferreira dos Santos	2.00	31	male	Complete Primary Education	DENGUE E OUTRAS DOENÇAS VIRAIS NE - A77	0.00	Solução o	10.00	Oral	Medicamentos<br>Dipirona Sódica 500 mg/ml Frasco 20 ml Solução oral<br>1 FRASCOS<br><br>Posologia:<br>20 gotas. a cada 6 horas. durante 5 dias	ST_medication	1	73.16	3	2	2	0	1	1	0	0	1	0	89.08	2	2	2	0	1	1	0	0	0	1	89.82	2	2	2	0	1	1	0	0	0	0	77.57	3.00	4.00	2.00	1.00	0.00	1.00	1.00	0.00	0.00	0.00
40	Juliana Santos Oliveira Silva Fonseca	1.00	14	female	Complete Primary Education	CONTRACEPÇÃO ORAL - W11	0.00	Pills	10.00	Oral	Medicamentos<br>Drospirenona + Etinilestradiol 3 mg + 0.02 mg Cartela Pills<br>6 CAIXAS<br><br>Posologia:<br>1 Pills. 1 vez ao dia. período indeterminado	LT_medication	0	81.60	2	2	2	1	0	1	0	0	1	0	93.04	4	1	2	1	0	1	0	0	0	0	94.23	2	1	1	1	0	1	0	0	0	0	89.41	2.00	1.00	2.00	1.00	0.00	1.00	0.00	0.00	1.00	0.00

41	Eduardo Pereira da Rocha Souza Almeida	2.00	30	male	High School	INSUFICIÊNCIA CARDÍACA - K77	0.00	Pills	10.00	Oral	Medicamentos<br>Enalapril. Maleato 20 mg Pills Pills<br>120 PillsS<br><br>Posologia:<br>1 Pills. 1 vez ao dia. período indeterminado	LT_medication	0	84.88	2	2	2	0	0	0	0	0	1	0	93.97	2	1	2	0	0	1	0	0	0	0	93.64	1	1	1	0	0	0	0	0	0	0	92.88	3.00	1.00	3.00	0.00	0.00	1.00	0.00	0.00	0.00	0.00
42	Adriana Silva Cardoso de Brito e Amaral	2.00	45	female	Complete Primary Education	EMBOLIA PULMONAR - K93	0.00	Solução ir	6.00	Intradermica	Medicamentos<br>Enoxaparina Sódica 40 mg Seringa 0.4 ml Solução injetável<br>6 SERINGAS<br><br>Posologia:<br>1 ampola. 1 vez ao dia. durante 6 dias	ST_medication	0	85.29	2	2	3	0	0	1	0	0	1	0	92.45	4	1	2	0	0	1	0	0	0	0	92.53	2	1	3	0	0	1	0	0	0	0	86.11	3.00	2.00	2.00	1.00	0.00	1.00	0.00	0.00	1.00	0.00
43	Mariana Oliveira Silva Santos Almeida	2.00	55	female	University. Diploma. Master's. Doctoral degree	DORES ABDOMINAIS. EPIGÁSTRICAS - D02	0.00	capsule	10.00	Oral	Medicamentos<br>Esomeprazol. Magnésio 40 mg Cápsula Cápsula<br>56 CÁPSULAS<br><br>Posologia:<br>1 cápsula. pela manhã. durante 1 mês	ST_medication	1	79.50	2	2	2	1	1	1	0	0	1	0	93.20	2	1	2	1	0	1	0	0	0	0	90.26	2	1	1	1	1	1	0	0	0	0	88.54	3.00	4.00	2.00	1.00	1.00	1.00	0.00	0.00	0.00	0.00
44	Lívia Almeida de Castro Yung	2.00	28	female	High School	INFECÇÕES QUE COMPLICAM A GRAVIDEZ - W71	0.00	Pills	10.00	Oral	Medicamentos<br>Espiramicina (1.5 mui) 500 mg Pills Pills<br>120 PillsS<br><br>Posologia:<br>2 Pillss. a cada 8 horas. durante 5 meses	ST_medication	0	81.87	4	3	2	0	0	0	0	1	1	1	94.75	4	2	2	0	0	1	1	0	0	0	92.51	1	1	1	0	0	0	0	0	0	0	91.92	3.00	2.00	5.00	1.00	0.00	1.00	0.00	0.00	0.00	0.00
45	Lívia Ribeiro dos Santos Oliveira Kimura	2.00	38	female	University. Diploma. Master's. Doctoral degree	SINAIS/SINTOMAS DA MENOPAUSA/ CLIMATÉRIO - X11	0.00	Gel	14.00	Transdermica	Medicamentos<br>Estradiol 0.75 mg Bisnaga 80 g Gel<br>3 BISNAGAS<br><br>Posologia:<br>1 aplicação. 1 vez ao dia. período indeterminado	LT_medication	0	84.12	2	2	1	0	1	0	0	0	1	0	94.08	4	1	2	0	0	0	0	0	1	0	94.44	3	2	2	1	1	1	0	0	0	0	91.20	4.00	1.00	2.00	1.00	0.00	0.00	1.00	0.00	0.00	0.00
46	Larissa Costa Mello Santos Silva	2.00	47	female	High School	SINAIS/SINTOMAS DA MENOPAUSA/ CLIMATÉRIO - X11	0.00	Creme	15.00	Vaginal	Medicamentos<br>Estriol 1 mg/g Bisnaga 50 g Creme vaginal<br>2 BISNAGAS<br><br>Posologia:<br>1 aplicação. pela noite. durante 3 meses	ST_medication	0	85.03	4	3	2	1	0	1	0	0	1	0	94.57	2	1	2	1	0	1	0	0	1	0	94.19	2	2	2	1	0	1	0	0	1	0	91.10	2.00	1.00	1.00	0.00	0.00	1.00	0.00	0.00	0.00	0.00
47	Ana Beatriz Silva Ferreira dos Santos Cho	2.00	54	female	Elementary Education 4th to 8th grades	OSTEOARTROSE DO JOELHO - L90	0.00	Pills	10.00	Oral	Medicamentos<br>Etodolaco 300 mg Pills Pills<br>21 PillsS<br><br>Posologia:<br>1 Pills. a cada 8 horas. durante 7 dias	ST_medication	1	84.05	2	2	2	0	0	0	0	0	1	0	93.59	2	1	2	0	0	0	0	0	0	0	90.73	1	1	1	0	0	0	0	0	0	0	90.69	1.00	4.00	2.00	0.00	0.00	1.00	0.00	0.00	0.00	0.00
48	Juliana Santos Almeida Araújo Ribeiro	3.00	77	female	High School	ARTRITE REUMATÓIDE/SEROPOSITIVA - L88	0.00	Pills	10.00	Oral	Medicamentos<br>Etoricoxibe 60 mg Pills Pills<br>3 PillsS<br><br>Posologia:<br>1 Pills. 1 vez ao dia. durante 3 dias	ST_medication	1	78.16	2	1	2	0	0	1	0	0	1	0	92.91	2	2	1	0	0	0	0	0	0	0	93.36	2	1	1	0	0	1	0	0	0	0	93.18	1.00	1.00	1.00	0.00	0.00	0.00	0.00	0.00	0.00	0.00
49	Ana Clara Ribeiro Silva Santos Souza	2.00	45	female	High School	CONVULSÕES/ATAQUES - N07	1.00	Pills	10.00	Oral	Medicamentos<br>Fenobarbital 100 mg Pills Pills<br>120 PillsS<br><br>Posologia:<br>1 Pills. a cada 12 horas. período indeterminado	LT_medication	0	82.28	4	2	2	0	0	0	0	0	1	0	92.07	2	2	2	0	0	0	0	0	1	0	92.82	2	1	2	0	0	0	0	0	1	0	94.00	5.00	2.00	2.00	1.00	0.00	0.00	0.00	0.00	0.00	0.00
50	Mariana Silva dos Santos Correa Lima	2.00	46	female	Elementary Education 1st to 4th grades	SECREÇÃO VAGINAL - X14	0.00	capsule	10.00	Oral	Medicamentos<br>Fluconazol 150 mg Cápsula Cápsula<br>2 CÁPSULAS<br><br>Posologia:<br>1 cápsula. 1 vez a cada 1 semana. durante 2 semanas	ST_medication	0	81.74	2	4	2	0	1	1	0	0	1	0	91.87	2	2	2	0	1	1	0	0	0	0	92.76	4	2	2	1	1	1	0	0	0	0	93.25	2.00	1.00	2.00	0.00	0.00	1.00	0.00	0.00	0.00	0.00
51	Amanda Souza Rocha Pereira Lima	2.00	22	female	High School	ANOREXIA NERVOSA. BULIMIA - P86	0.00	capsule	10.00	Oral	Medicamentos<br>Fluoxetina. Cloridrato 20 mg Cápsula Cápsula<br>90 CÁPSULAS<br><br>Posologia:<br>1 cápsula. pela manhã. durante 3 meses	ST_medication	0	83.53	2	2	2	0	0	0	0	0	1	0	92.53	2	1	2	0	0	0	0	0	0	0	93.04	3	2	2	0	0	1	0	0	1	0	94.13	2.00	1.00	1.00	0.00	0.00	1.00	0.00	0.00	0.00	0.00
52	Júlia Andrade de Almeida Menezes Ferreira	2.00	28	female	High School	RINITE ALÉRGICA - R97	0.00	Aerosol	1.00	Inalatoria nasal	Medicamentos<br>Fluticasona. Propionato 50 mcg Frasco 10 ml Spray nasal<br>120 DOSES<br><br>Posologia:<br>2 aplicações. 1 vez ao dia. durante 5 dias	ST_medication	0	83.32	1	2	2	0	0	0	0	0	0	0	93.45	2	1	1	0	0	0	0	0	0	0	92.55	2	2	1	0	0	1	0	0	0	0	91.98	5.00	5.00	2.00	1.00	0.00	1.00	0.00	1.00	0.00	0.00

53	Carlos Eduardo Silva Santos Ornellas Sampaio	1.00	19	male	Complete Primary Education	ALTERAÇÃO NO METABOLISMO DOS LÍPIDOS - T93	0.00	Pills	10.00	Oral	Medicamentos<br>Ezetimiba 10 mg Pills Pills<br>28 PillsS<br><br>Posologia:<br>1 Pills. 1 vez ao dia. período indeterminado	LT_medication	0	84.69	2	2	2	0	1	1	0	0	1	0	91.98	3	1	2	0	0	1	0	0	0	0	89.68	3	2	1	0	0	1	0	0	0	0	92.38	1.00	4.00	2.00	0.00	0.00	1.00	0.00	0.00	0.00	0.00
54	Felipe Souza Melo Pereira Yang	2.00	39	male	High School	URETRITE - U72	0.00	Granulad	10.00	Oral	Medicamentos<br>Fosfomicina Trometamol 3 g Envelope 8 g Granulado<br>1 ENVELOPES<br><br>Posologia:<br>1 envelope. 1 vez ao dia. durante 1 dia	ST_medication	0	85.93	1	2	2	0	0	0	0	0	1	0	95.51	2	1	2	0	1	1	0	0	0	0	94.13	4	2	2	0	0	1	0	0	0	0	95.60	2.00	2.00	2.00	1.00	0.00	1.00	0.00	0.00	0.00	0.00
55	Luís Ferreira Rocha de Almeida Santos	2.00	29	male	No schooling	DOR GENERALIZADA /MÚLTIPLA - A01	1.00	Pills	10.00	Oral	Medicamentos<br>Gabapentina 600 mg Pills Pills<br>120 PillsS<br><br>Posologia:<br>1 Pills. a cada 12 horas. período indeterminado	LT_medication	0	80.70	2	2	2	0	0	1	0	0	0	0	89.96	2	1	2	0	1	1	0	0	0	0	92.87	1	1	1	0	0	0	0	0	0	0	94.48	2.00	1.00	2.00	0.00	0.00	0.00	0.00	0.00	0.00	0.00
56	Lúcio Souza Albuquerque Kimura Silva	2.00	52	male	High School	DIABETES 2 INSULINO-DEPENDENTE - T90	0.00	Pills	10.00	Oral	Medicamentos<br>Gliclazida 30 mg Pills Pills de liberação prolongada<br>90 PillsS<br><br>Posologia:<br>1 Pills. pela manhã. período indeterminado	LT_medication	0	80.27	4	2	2	0	0	1	0	0	1	0	93.21	2	2	2	0	0	1	0	0	0	0	95.15	2	1	2	0	0	0	0	0	0	0	92.56	3.00	1.00	2.00	1.00	0.00	0.00	0.00	0.00	1.00	0.00
57	Carolina Silva dos Santos Gomes Lima	3.00	64	female	No schooling	AZIA/ QUEIMAÇÃO - D03	0.00	Suspensã	10.00	Oral	Medicamentos<br>Hidróxido de Alumínio + Hidróxido de Magnésio 60 + 40 mg/ml Frasco 240 ml Suspensão oral<br>1 FRASCOS<br><br>Posologia:<br>10 ml (mililitros). 3 vezes ao dia. durante 5 dias	ST_medication	1	81.87	5	4	2	1	1	1	0	0	1	0	89.43	4	1	2	1	0	1	0	1	0	0	92.48	3	2	2	1	0	0	0	0	1	0	90.41	1.00	1.00	2.00	0.00	0.00	0.00	0.00	0.00	0.00	0.00
58	Leonardo Bento Pereira de Oliveira	2.00	42	male	High School	AZIA/ QUEIMAÇÃO - D03	0.00	Pills	10.00	Oral	Medicamentos<br>Hidróxido de Alumínio 300 mg Pills Pills<br>56 PillsS<br><br>Posologia:<br>2 Pillss. 4 vezes ao dia. durante 7 dias	ST_medication	1	80.35	4	2	2	1	0	1	0	1	1	0	88.57	3	2	2	1	1	1	0	0	1	0	91.99	2	2	2	1	1	1	0	0	0	0	94.47	1.00	4.00	2.00	0.00	0.00	1.00	0.00	0.00	0.00	0.00
59	Júlia Santos Ferreira da Cunha Chin	2.00	20	female	University. Diploma. Master's. Doctoral degree	ENXAQUECA - N89	0.00	Pills	10.00	Oral	Medicamentos<br>Ibuprofeno 400 mg Pills Pills<br>30 PillsS<br><br>Posologia:<br>1 Pills. a cada 12 horas. durante 15 dias	ST_medication	1	85.00	4	3	2	1	1	1	0	0	1	0	87.43	3	2	2	1	1	1	0	0	0	0	85.77	4	2	2	1	0	1	0	0	0	0	96.40	3.00	1.00	1.00	0.00	0.00	1.00	0.00	0.00	0.00	0.00
60	João Carlos dos Santos Pereira Silva	2.00	39	male	Complete Primary Education	CEFALÉIA - N01	0.00	Pills	10.00	Oral	Medicamentos<br>Ibuprofeno 600 mg Pills Pills<br>15 PillsS<br><br>Posologia:<br>1 Pills. a cada 8 horas. durante 5 dias	ST_medication	1	85.20	2	2	2	0	0	1	0	0	1	0	93.65	2	2	1	0	1	0	0	0	0	0	89.33	2	1	1	0	0	1	0	0	0	0	89.84	3.00	1.00	2.00	1.00	1.00	1.00	1.00	0.00	1.00	0.00
61	Carlos Pereira Santos da Costa Silva	1.00	18	male	Complete Primary Education	DOENÇAS DO FÍGADO /NE - D97	0.00	Pills	10.00	Oral	Medicamentos<br>Indapamida 1.5 mg Pills Pills<br>30 PillsS<br><br>Posologia:<br>1 Pills. pela manhã. durante 30 dias	ST_medication	0	84.79	2	2	2	0	1	0	0	0	1	0	96.61	3	1	2	0	0	1	0	0	0	0	95.65	2	1	2	0	0	1	0	0	0	0	92.96	4.00	2.00	2.00	1.00	0.00	0.00	1.00	0.00	0.00	0.00
62	Lucas Oliveira Santos Medeiros Lima	3.00	82	male	Elementary Education 4th to 8th grades	DIABETES INSULINO-DEPENDENTE - T89	0.00	Suspensã	6.00	Intradermica	Medicamentos<br>Insulina Humana Nph 100 ui/ml Frasco-ampola 10 ml Suspensão injetável<br>10 FRASCOS-AMPOLAS<br><br>Posologia:<br>10 ui (unidade internacional). 3 vezes ao dia. período indeterminado	LT_medication	0	83.71	2	2	2	0	0	1	0	0	1	0	91.93	2	1	1	0	0	1	0	0	0	0	91.27	1	1	2	0	0	0	0	0	0	0	90.42	2.00	2.00	2.00	0.00	0.00	1.00	0.00	0.00	0.00	0.00
63	Júlia Santos Pereira Chaves Sampaio	1.00	17	female	Complete Primary Education	PEDICULOSE/OUTRAS INFECÇÕES DA PELE - S73	0.00	Pills	10.00	Oral	Medicamentos<br>Ivermectina 6 mg Pills Pills<br>8 PillsS<br><br>Posologia:<br>4 Pillss. 1 vez a cada 14 dias . durante 14 dias	ST_medication	0	76.12	4	2	1	1	1	1	1	0	1	0	88.87	4	2	1	0	1	1	1	0	0	0	88.58	2	2	1	1	1	0	1	0	1	0	90.59	4.00	1.00	1.00	1.00	0.00	1.00	0.00	0.00	0.00	0.00
64	Lucas Pereira dos Santos da Conceição	1.00	15	male	Complete Primary Education	SINUSITE CRÔNICA/AGUDA - R75	0.00	Pills	10.00	Oral	Medicamentos<br>Levofloxacino 500 mg Pills Pills<br>10 PillsS<br><br>Posologia:<br>1 Pills. a cada 1 dia. durante 10 dias	ST_medication	0	78.59	4	4	2	0	0	0	0	0	1	0	93.28	2	1	2	0	0	0	0	0	1	0	91.96	4	1	2	0	0	0	0	0	1	0	92.93	3.00	1.00	2.00	0.00	0.00	0.00	0.00	0.00	1.00	0.00

65	Lívia Santos de Oliveira Silva	2.00	26	female	High School	CONTRACEPÇÃO PÓS-COITAL - W10	0.00	Pills	10.00	Oral	Medicamentos<br>Levonorgestrel 1.5 mg Pills Pills<br>1 PillsS<br><br>Posologia:<br>1 Pills. dose única. durante 1 dia	ST_medication	0	86.39	2	1	2	0	0	0	0	0	1	0	96.04	4	1	2	0	0	1	0	0	0	0	92.06	2	1	1	0	0	1	0	0	0	0	90.00	2.00	1.00	2.00	0.00	0.00	0.00	0.00	0.00	0.00	0.00
66	Renata Seixas Barbosa Amorim	1.00	18	female	High School	HIPOTIROIDISMO/MIXEDEMA - T86	0.00	Pills	10.00	Oral	Medicamentos<br>Levotiroxina Sódica 100 mcg Pills Pills<br>30 PillsS<br><br>Posologia:<br>1 Pills. pela manhã. período indeterminado	LT_medication	0	85.96	3	2	2	1	0	1	0	0	1	0	96.97	2	1	2	1	0	1	0	0	0	0	90.95	1	1	1	0	0	0	0	0	0	0	93.30	1.00	4.00	2.00	0.00	0.00	1.00	0.00	0.00	0.00	0.00
67	Carolina Costa Ribeiro da Silva	2.00	36	female	Complete Primary Education	HIPERTENSÃO SEM COMPLICAÇÕES - K86	0.00	Pills	10.00	Oral	Medicamentos<br>Losartana Potássica 50 mg Pills Pills<br>180 PillsS<br><br>Posologia:<br>1 Pills. 1 vez ao dia. período indeterminado	LT_medication	0	83.52	3	3	2	0	1	0	0	0	1	0	98.60	4	1	2	0	0	1	0	0	0	0	92.35	1	1	1	0	0	0	0	0	0	0	85.76	2.00	2.00	1.00	0.00	0.00	1.00	0.00	0.00	0.00	0.00
68	Guilherme Silva Santos Ferreira	2.00	29	male	Complete Primary Education	OBESIDADE - T82	0.00	Pills	10.00	Oral	Medicamentos<br>Metformina. Cloridrato 500 mg Pills Pills de liberação controlada<br>90 PillsS<br><br>Posologia:<br>1 Pills. pela manhã. período indeterminado	LT_medication	0	88.13	2	2	2	0	0	1	0	0	1	0	94.96	2	2	1	0	0	1	0	0	0	0	95.28	1	1	1	0	0	0	0	0	0	0	90.53	4.00	4.00	2.00	0.00	0.00	1.00	0.00	0.00	0.00	0.00
69	André Martins Silva Almeida Souza	2.00	46	male	High School	DERMATITE/ECZEMA ATÓPICO - S87	0.00	Creme	3.00	Dermatologica	Medicamentos<br>Metilprednisolona. Aceponato 1 mg/g Bisnaga 15 g Creme<br>1 BISNAGAS<br><br>Posologia:<br>2 aplicações. 2 vezes ao dia. durante 5 dias	ST_medication	0	80.62	2	2	2	0	0	0	0	1	0	0	88.62	2	1	2	0	0	0	0	0	0	0	87.13	2	1	1	0	0	0	0	0	0	0	92.88	2.00	1.00	2.00	0.00	0.00	0.00	1.00	0.00	1.00	0.00
70	Lucas da Silva Santos Ferreira Araújo	3.00	60	male	University. Diploma. Master's. Doctoral degree	URETRITE - U72	0.00	Pills	10.00	Oral	Medicamentos<br>Metronidazol 250 mg Pills Pills<br>40 PillsS<br><br>Posologia:<br>2 Pillss. a cada 12 horas. durante 10 dias	ST_medication	0	80.85	2	2	2	0	0	0	0	0	1	0	93.15	2	2	2	0	0	0	0	0	1	0	94.09	3	2	2	1	0	0	0	0	1	0	94.13	3.00	2.00	2.00	0.00	0.00	0.00	0.00	0.00	1.00	0.00
71	Lucas Pereira dos Santos Souza Silva	2.00	38	male	Elementary Education 4th to 8th grades	DERMATOFITOSE - S74	0.00	Creme	3.00	Dermatologica	Medicamentos<br>Miconazol. Nitrato 2 % Bisnaga 80 g Creme<br>1 BISNAGAS<br><br>Posologia:<br>1 aplicação. pela noite. durante 10 dias	ST_medication	0	78.28	2	2	2	0	0	0	0	0	1	0	95.58	3	1	1	0	0	1	0	0	0	0	91.19	2	1	2	0	0	1	0	0	0	0	92.56	1.00	4.00	2.00	0.00	0.00	1.00	0.00	0.00	0.00	0.00
72	Isabela da Silva De Andrade Tanaka	1.00	14	female	No schooling	DORES MUSCULARES - L18	0.00	Pills	10.00	Oral	Medicamentos<br>Naproxeno Sódico 500 mg Pills Pills<br>10 PillsS<br><br>Posologia:<br>1 Pills. a cada 1 dia. durante 10 dias	ST_medication	1	79.19	2	2	1	1	1	0	0	0	1	0	87.04	2	2	1	1	1	0	0	0	0	0	91.36	4	2	1	1	1	0	0	0	0	0	92.89	2.00	1.00	1.00	0.00	0.00	0.00	0.00	0.00	0.00	0.00
73	Lívia Ribeiro Almeida Santos Pacheco	2.00	41	female	High School	CISTITE/OUTRA INFECÇÃO URINÁRIA - U71	0.00	Pills	10.00	Oral	Medicamentos<br>Nitrofurantoína 100 mg Pills Pills<br>28 PillsS<br><br>Posologia:<br>1 Pills. a cada 6 horas. durante 7 dias	ST_medication	0	86.30	3	4	2	0	0	0	0	0	1	0	94.19	4	2	2	1	0	0	0	0	1	0	93.24	4	1	1	0	0	0	0	0	0	0	94.32	4.00	1.00	1.00	1.00	1.00	0.00	0.00	0.00	0.00	1.00
74	Luiz Henrique Castro Alves Batista Carvalho	2.00	40	male	High School	CONJUNTIVITE INFECCIOSA - F70	0.00	Solução o	9.00	Oftalmica	Medicamentos<br>Ofloxacino 3 % Frasco 5 ml Solução oftálmica<br>1 FRASCOS<br><br>Posologia:<br>1 gota. a cada 4 horas. durante 10 dias	ST_medication	0	80.33	2	2	2	0	1	1	0	1	1	0	89.23	2	2	2	0	1	1	0	1	0	0	90.93	2	2	2	0	1	0	0	0	1	0	91.99	2.00	1.00	1.00	0.00	0.00	0.00	0.00	0.00	0.00	0.00
75	Ana Beatriz Ferreira da Silva Souza Menezes	3.00	75	female	High School	DENGUE E OUTRAS DOENÇAS VIRAIS NE - A77	0.00	Pills	13.00	Sublingual	Medicamentos<br>Ondansetrona. Cloridrato 8 mg Pills Pills<br>21 PillsS<br><br>Posologia:<br>1 Pills. a cada 8 horas . durante 7 dias	ST_medication	1	80.11	3	2	2	1	0	1	0	0	1	0	88.94	3	2	2	1	0	1	0	0	1	0	91.23	3	4	2	1	0	1	0	0	1	0	95.22	2.00	2.00	2.00	0.00	0.00	1.00	0.00	0.00	0.00	0.00
76	Leonardo Alves de Moraes Silva	2.00	33	male	No schooling	INCONTINÊNCIA URINÁRIA - U04	0.00	Pills	10.00	Oral	Medicamentos<br>Oxibutinina. Cloridrato 5 mg Pills Pills<br>360 PillsS<br><br>Posologia:<br>1 Pills. a cada 12 horas. período indeterminado	LT_medication	0	72.83	2	1	2	0	0	0	0	0	1	0	91.13	3	3	2	0	0	0	0	0	1	0	93.49	4	1	2	0	0	0	0	0	1	0	90.51	3.00	2.00	2.00	1.00	0.00	1.00	0.00	0.00	0.00	1.00

77	Lívia da Silva Melo Andrade Kimura	2.00	44	female	Adult literacy course	ALTERAÇÕES FUNCIONAIS ESTÔMAGO - D87	0.00	Pills	10.00	Oral	Medicamentos<br>Pantoprazol 40 mg Pills Pills<br>56 PillsS<br><br>Posologia:<br>1 Pills. pela manhã. durante 8 semanas	ST_medication	0	91.28	2	3	3	0	0	0	0	0	1	0	94.04	1	1	2	0	0	0	0	0	0	0	94.13	3	1	2	0	0	0	0	0	1	0	90.63	4.00	1.00	2.00	1.00	0.00	1.00	0.00	0.00	1.00	0.00
78	José Carlos Silva Santos Neto	2.00	34	male	High School	SINAIS/SINTOMAS DO JOELHO - L15	0.00	Pills	10.00	Oral	Medicamentos<br>Paracetamol + Codeína. Fosfato 500 mg + 30 mg Pills Pills<br>20 PillsS<br><br>Posologia:<br>1 Pills. a cada 4 horas. durante 5 dias	ST_medication	1	74.03	1	1	2	0	0	0	0	0	1	0	90.34	4	1	2	0	0	0	0	0	1	0	89.83	1	1	2	0	0	0	0	0	0	0	91.48	4.00	1.00	1.00	1.00	0.00	1.00	0.00	0.00	0.00	0.00
79	Leonardo Santos Oliveira Castanho Silva	2.00	19	male	University. Diploma. Master's. Doctoral degree	DENGUE E OUTRAS DOENÇAS VIRAIS NE - A77	0.00	Pills	10.00	Oral	Medicamentos<br>Paracetamol 750 mg Pills Pills<br>20 PillsS<br><br>Posologia:<br>1 Pills. a cada 6 horas. durante 5 dias	ST_medication	1	72.98	2	2	2	0	0	0	0	0	1	0	90.30	4	2	2	1	0	1	0	0	0	0	89.49	2	1	1	1	0	0	0	0	0	0	74.27	4.00	1.00	1.00	1.00	0.00	1.00	0.00	0.00	0.00	0.00
80	Luiz Carlos Silva dos Santos Ribeiro	2.00	34	male	University. Diploma. Master's. Doctoral degree	PERTURBAÇÃO DO SONO - P06	0.00	Pills	10.00	Oral	Medicamentos<br>Passiflora Incarnata 360 mg Pills Pills<br>30 PillsS<br><br>Posologia:<br>1 Pills. a cada 12 horas. durante 30 dias	ST_medication	1	83.92	2	2	2	0	1	0	0	0	1	0	96.90	2	2	1	0	1	0	0	0	0	0	95.88	2	2	1	0	1	0	0	0	0	0	94.22	1.00	4.00	2.00	0.00	0.00	0.00	0.00	0.00	0.00	0.00
81	Leonardo Oliveira Dias Medeiros Santos	3.00	67	male	Elementary Education 4th to 8th grades	INSUFICIÊNCIA CARDÍACA - K77	0.00	Pills	10.00	Oral	Medicamentos<br>Perindopril + Indapamida 4 + 1.25 mg Pills Pills<br>30 PillsS<br><br>Posologia:<br>1 Pills. 1 vez ao dia. período indeterminado	LT_medication	0	75.48	4	4	2	1	0	0	0	0	1	0	93.04	3	2	2	1	0	1	0	0	1	0	93.90	4	1	2	1	0	0	0	0	1	0	93.85	4.00	4.00	2.00	0.00	0.00	0.00	1.00	0.00	0.00	0.00
82	Júlia Santos Silva Sousa Carvalho	3.00	77	female	No schooling	HIPERTENSÃO SEM COMPLICAÇÕES - K86	0.00	Pills	10.00	Oral	Medicamentos<br>Perindopril 4 mg Pills Pills<br>60 PillsS<br><br>Posologia:<br>2 Pillss. 1 vez ao dia. período indeterminado	LT_medication	0	77.16	2	2	2	0	1	0	0	0	1	0	92.10	2	2	1	0	1	1	0	0	0	0	91.61	2	2	1	0	1	0	0	0	0	0	91.89	3.00	1.00	2.00	1.00	0.00	1.00	0.00	0.00	0.00	0.00
83	Larissa dos Santos Silva Nobrega Okada	2.00	55	female	High School	HEMORRÓIDAS - K96	0.00	Pomada	12.00	Retal	Medicamentos<br>Policresuleno + Cinchocaína. Cloridrato 50 + 10 mg/g Bisnaga 30 g Pomada retal<br>1 BISNAGAS<br><br>Posologia:<br>1 aplicação. 2 vezes ao dia. durante 7 dias	ST_medication	0	83.81	4	1	2	1	0	1	0	0	1	0	84.33	5	4	2	1	0	1	0	0	1	0	92.13	4	2	2	1	0	1	0	0	1	0	91.72	2.00	2.00	1.00	0.00	0.00	0.00	0.00	0.00	1.00	0.00
84	Leonardo Silva Ribeiro dos Santos	2.00	44	male	Elementary Education 4th to 8th grades	ASMA - R96	0.00	Pills	10.00	Oral	Medicamentos<br>Prednisona 20 mg Pills Pills<br>10 PillsS<br><br>Posologia:<br>2 Pillss. 1 vez ao dia. durante 5 dias	ST_medication	0	70.14	2	2	1	0	1	1	0	0	1	0	87.80	2	2	1	0	1	1	0	0	0	0	89.91	1	2	1	0	0	0	0	0	0	0	91.78	2.00	2.00	2.00	0.00	0.00	0.00	0.00	0.00	0.00	0.00
85	Ana Carolina Silva Medeiros Menezes Guimarães	1.00	15	female	University. Diploma. Master's. Doctoral degree	SINAIS/SINTOMAS DA MENOPAUSA/ CLIMATÉRIO - X11	0.00	capsule	15.00	Vaginal	Medicamentos<br>Progesterona 100 mg Cápsula Cápsula<br>30 CÁPSULAS<br><br>Posologia:<br>1 cápsula. pela noite. período indeterminado	LT_medication	0	76.07	1	2	2	0	0	0	0	0	1	0	90.71	2	1	1	0	0	1	0	0	0	0	92.59	1	1	1	0	0	0	0	0	0	0	93.54	2.00	1.00	1.00	0.00	0.00	0.00	0.00	0.00	0.00	0.00
86	Lucas Santos Oliveira Lima Guimarães	2.00	47	male	High School	HIPERTIROIDISMO/TIREOTOXICOSE - T85	0.00	Pills	10.00	Oral	Medicamentos<br>Propiltiouracila 100 mg Pills Pills<br>360 PillsS<br><br>Posologia:<br>1 Pills. a cada 12 horas. período indeterminado	LT_medication	0	72.22	2	2	2	0	1	0	0	0	1	0	87.53	2	2	2	0	1	0	0	0	0	0	93.52	2	1	2	0	1	0	0	0	0	0	94.90	4.00	4.00	2.00	0.00	0.00	1.00	0.00	0.00	0.00	0.00
87	Larissa Pereira da Silva Pinto Nakagawa	2.00	35	female	University. Diploma. Master's. Doctoral degree	TROMBOSE/ACIDENTE VASCULAR CEREBRAL - K90	0.00	Pills	10.00	Oral	Medicamentos<br>Rivaroxabana 20 mg Pills Pills<br>120 PillsS<br><br>Posologia:<br>1 Pills. 1 vez ao dia. durante 120 dias	ST_medication	0	79.96	2	1	2	0	0	0	0	0	0	0	89.97	1	2	2	0	0	0	0	0	0	0	92.18	2	1	1	0	0	0	0	0	1	0	93.70	4.00	4.00	2.00	0.00	0.00	1.00	0.00	0.00	0.00	0.00
88	João Santos de Oliveira Kimura	2.00	48	male	Elementary Education 1st to 4th grades	DIARREIA - D11	0.00	Po para s	10.00	Oral	Medicamentos<br>Sais para Reidratação Oral (Nacl 3.5 g + Glicose 20 g + Citrato Na 2.9 g + Kcl 1.5 g) Envelope 8.5 g Pó para solução<br>6 ENVELOPES<br><br>Posologia:<br>1 envelope. a cada 12 horas. durante 3 dias	ST_medication	0	74.43	4	1	2	1	0	0	0	0	1	0	79.87	3	2	2	1	0	1	0	0	1	0	77.64	2	4	2	1	0	1	0	0	1	0	85.16	2.00	1.00	2.00	1.00	0.00	1.00	0.00	0.00	0.00	0.00

89	Ana Luísa Cardoso Silva Mendes Santos	1.00	14	female	Elementary Education 4th to 8th grades	ASMA - R96	0.00	Aerosol	5.00	Inalatória por vi	Medicamentos<br>Salbutamol. Sulfato 100 mcg/dose Frasco 200 doses Aerosol<br>1 DOSES<br><br>Posologia:<br>2 aplicações. a cada 6 horas. período indeterminado	LT_medication	0	86.47	2	2	2	0	0	1	0	0	0	0	89.51	2	2	2	0	0	1	0	0	0	0	90.42	2	1	2	0	0	0	0	0	0	0	95.20	2.00	1.00	2.00	0.00	0.00	0.00	0.00	0.00	0.00	0.00
90	Maurício Santos da Costa Mendes	2.00	25	male	High School	MEDO DE DISFUNÇÃO SEXUAL MASCULINA - Y24	0.00	Pills	10.00	Oral	Medicamentos<br>Sildenafila. Citrato 25 mg Pills Pills<br>8 PillsS<br><br>Posologia:<br>1 Pills. dose única. durante 1 dia	ST_medication	0	80.82	2	2	2	1	1	1	0	0	1	0	90.75	2	2	1	1	1	1	0	0	0	0	91.86	2	2	1	0	0	1	0	0	0	0	92.96	2.00	2.00	5.00	0.00	1.00	0.00	0.00	0.00	0.00	1.00
91	Carlos Eduardo Santos Silva Moura Costa	2.00	48	male	High School	ALTERAÇÃO NO METABOLISMO DOS LÍPIDOS - T93	0.00	Pills	10.00	Oral	Medicamentos<br>Sinvastatina 20 mg Pills Pills<br>120 PillsS<br><br>Posologia:<br>1 Pills. pela manhã. durante 4 meses	ST_medication	0	82.38	4	1	2	0	0	0	0	0	1	0	92.86	2	1	2	0	0	0	0	0	1	0	95.30	4	1	2	0	0	0	0	0	1	0	94.82	2.00	2.00	2.00	0.00	0.00	0.00	0.00	0.00	0.00	0.00
92	Leonardo Souza Rocha de Almeida Lima	3.00	90	male	University. Diploma. Master's. Doctoral degree	DIABETES 2 INSULINO-DEPENDENTE - T90	0.00	Pills	10.00	Oral	Medicamentos<br>Sitagliptina. Fosfato + Metformina. Cloridrato 50 + 1000 mg Pills Pills<br>60 PillsS<br><br>Posologia:<br>1 Pills. 2 vezes ao dia. durante 30 dias	ST_medication	0	72.75	1	1	2	0	0	0	0	0	1	0	91.44	2	1	2	0	0	0	0	0	1	0	93.05	2	1	2	1	0	0	0	0	1	0	95.48	2.00	2.00	1.00	0.00	0.00	0.00	0.00	0.00	1.00	0.00
93	Livia Silva Pereira Chu Soares	3.00	68	female	High School	CEFALÉIA - N01	0.00	Pills	10.00	Oral	Medicamentos<br>Succinato de Sumatriptano 50 mg Pills Pills<br>4 PillsS<br><br>Posologia:<br>1 Pills. 1 vez ao dia. durante 4 dias	ST_medication	1	84.15	2	2	2	0	1	1	0	0	1	0	89.61	2	2	1	0	1	1	0	0	1	0	90.31	2	2	1	0	1	1	0	0	0	0	91.27	2.00	2.00	2.00	1.00	0.00	1.00	0.00	0.00	0.00	0.00
94	Lívia Santos Pereira Ribeiro Silva	2.00	37	female	Complete Primary Education	ÚLCERA PÉPTICA. OUTRA - D86	0.00	Pills	10.00	Oral	Medicamentos<br>Sucralfato 1 g Pills Pills<br>60 PillsS<br><br>Posologia:<br>1 Pills. 3 vezes ao dia. durante 20 dias	ST_medication	0	84.57	2	2	2	0	0	1	0	1	0	0	93.24	2	2	1	0	0	1	0	0	0	0	94.38	2	2	2	0	1	0	0	0	0	0	90.24	4.00	5.00	2.00	0.00	1.00	0.00	1.00	0.00	0.00	1.00
95	Lara Ribeiro da Silva Zhang de Sousa	2.00	50	female	High School	ANEMIA POR DEFICIÊNCIA FERRO - B80	0.00	Pills	10.00	Oral	Medicamentos<br>Sulfato Ferroso (60 mg de Ferro Elementar) 300 mg Pills Pills<br>180 PillsS<br><br>Posologia:<br>2 Pillss. 1 vez ao dia. durante 3 meses	ST_medication	0	75.87	4	4	2	1	0	1	0	1	1	0	91.18	2	1	2	1	0	1	0	0	1	0	92.86	5	1	2	1	0	1	0	0	1	0	92.75	4.00	1.00	2.00	1.00	1.00	0.00	0.00	0.00	0.00	0.00
96	André Ferreira dos Santos Silva	2.00	21	male	High School	SINAIS/SINTOMAS DAS ARTICULAÇÕES NE - L20	0.00	Pomada	8.00	Local	Medicamentos<br>Symphitum Sp L. (Confrei) 5 % Bisnaga 50 g Pomada<br>1 BISNAGAS<br><br>Posologia:<br>1 aplicação. 4 vezes ao dia. durante 10 dias	ST_medication	1	87.87	4	1	2	0	0	0	0	0	1	0	89.11	2	1	2	1	0	1	0	0	1	0	87.98	4	1	2	1	0	0	0	0	1	0	83.12	2.00	1.00	2.00	0.00	0.00	0.00	0.00	0.00	0.00	0.00
97	Leonardo Almeida Barros dos Santos	1.00	16	male	High School	DERMATITE/ECZEMA ATÓPICO - S87	0.00	Pomada	3.00	Dermatologica	Medicamentos<br>Tacrolimo 1 mg/g Bisnaga 10 g Pomada<br>1 BISNAGAS<br><br>Posologia:<br>1 aplicação. 2 vezes ao dia. durante 14 dias	ST_medication	0	80.24	2	2	2	0	1	1	0	0	1	0	93.07	2	2	2	0	1	1	0	0	0	0	95.65	2	2	2	0	1	1	0	0	0	0	91.84	2.00	1.00	1.00	1.00	0.00	1.00	0.00	0.00	0.00	0.00
98	Luciana Soares Oliveira Santos Kumagai	3.00	60	female	High School	SINAIS/SINTOMAS DA REGIÃO LOMBAR - L03	0.00	Pills	10.00	Oral	Medicamentos<br>Tramadol. Cloridrato 100 mg Pills Pills<br>12 CÁPSULAS<br><br>Posologia:<br>1 cápsula. a cada 8 horas. durante 4 dias	ST_medication	1	79.75	4	1	2	1	0	0	0	0	1	0	86.40	2	2	2	1	0	0	0	0	1	0	84.94	4	1	2	1	0	1	0	0	1	0	93.02	3.00	1.00	2.00	0.00	0.00	1.00	0.00	1.00	1.00	0.00
99	Juliana Pereira dos Santos Ogawa Lima	2.00	46	female	University. Diploma. Master's. Doctoral degree	PARKINSONISMO - N87	0.00	Pills	10.00	Oral	Medicamentos<br>Triexifenidil. Cloridrato 2 mg Pills Pills<br>90 PillsS<br><br>Posologia:<br>1 Pills. 3 vezes ao dia. período indeterminado	LT_medication	0	76.26	2	2	2	0	1	0	0	0	1	0	89.87	2	2	1	0	1	1	0	0	1	0	90.44	2	2	1	0	1	1	0	0	0	0	90.43	1.00	1.00	1.00	0.00	0.00	0.00	0.00	0.00	0.00	0.00
100	Juliana Cardoso de Oliveira Nascimento	3.00	65	female	High School	HERPES ZOSTER - S70	0.00	Pills	10.00	Oral	Medicamentos<br>Valaciclovir. Cloridrato 500 mg Pills Pills<br>42 PillsS<br><br>Posologia:<br>2 Pillss de 500mg. a cada 8 horas. durante 7 dias	ST_medication	0	81.56	2	2	1	0	1	1	0	0	1	0	92.67	2	2	1	0	1	0	0	0	0	0	100.0C	4	2	1	1	1	1	1	0	0	0	92.37	2.00	2.00	2.00		0.00	0.00	0.00	0.00	0.00	0.00

101	Luiz Carlos Pereira de Castro Silva	2.00	23	male	Complete Primary Education	TROMBOSE/ACIDENTE VASCULAR CEREBRAL - K90	0.00	Pills	10.00	Oral	Medicamentos<br>Varfarina Sódica 5 mg Pills Pills<br>180 PillsS<br><br>Posologia:<br>1 Pills. 1 vez ao dia. período indeterminado	LT_medication	0	83.03	1	1	2	0	0	0	0	0	1	0	92.09	2	2	2	0	0	1	0	0	1	0	95.15	4	1	2	0	0	0	0	0	1	0	91.12	4.00	1.00	5.00	1.00	0.00	1.00	0.00	0.00	0.00	1.00
102	Felipe Santos Melo Silva Costa	2.00	46	male	High School	DIABETES 2 INSULINO-DEPENDENTE - T90	0.00	Pills	10.00	Oral	Medicamentos<br>Vildagliptina + Metformina. Cloridrato 50 + 1000 mg Pills Pills<br>90 PillsS<br><br>Posologia:<br>1 Pills. pela manhã. período indeterminado	LT_medication	0	83.79	2	2	2	0	1	0	0	0	1	0	92.66	2	2	2	0	1	0	0	0	0	0	93.93	2	2	1	0	1	0	0	0	0	0	86.47	2.00	4.00	2.00	1.00	0.00	1.00	0.00	0.00	0.00	0.00
103	Leonardo dos Santos Silva Barcelos	2.00	37	male	High School	TRISTEZA/ SENSAÇÃO DE DEPRESSÃO - P03	1.00	Pills	10.00	Oral	Medicamentos<br>Vortioxetina. Bromidato 10 mg Pills Pills<br>90 PillsS<br><br>Posologia:<br>1 cápsula. a cada 1 dia. durante 3 meses	ST_medication	0	71.76	2	2	2	0	0	0	0	0	1	0	88.62	2	2	1	0	0	1	0	0	0	0	90.24	1	1	1	0	0	0	0	0	0	0	94.30	1.00	1.00	1.00	0.00	0.00	0.00	0.00	0.00	0.00	0.00
104	João Gabriel Silva Ribeiro Almeida Gomes	2.00	29	male	High School	GLAUCOMA - F93	0.00	Pills	10.00	Oral	Medicamentos<br>Acetazolamida 250 mg Pills Pills<br>30 PillsS<br><br>Posologia:<br>1 Pills. a cada 1 dia. periodo indeterminado	LT_medication	0	80.75	2	4	2	0	0	0	0	0	1	0	92.67	2	2	1	0	0	0	0	0	1	0	88.00	1	1	1	0	0	0	0	0	0	0	92.44	3.00	2.00	2.00	1.00	1.00	0.00	0.00	0.00	0.00	0.00
