# Supplementary material for: Evaluating Large Language Model–Supported Instructions for Medication Use: First Steps Toward a Comprehensive Model
Source: Mayo Clin Proc Digit Health. 2024 Oct 19;2(4):632–44. doi: 10.1016/j.mcpdig.2024.09.006 (PMC11638470; doi:10.1016/j.mcpdig.2024.09.006)
Supplement: Supplemental Material [file mmc2.pdf]

Evaluating LLM-supported instructions for medication use: first steps towards a comprehensive model

Supplementary file 1 - Retrieval reports about best practices for safe use of medication, based on an Internet search, on May 2024.

1. BRASIL. Ministério da Saúde. Secretaria de Atenção Especializada à Saúde. Portaria SAES/MS nº 50, de 9 de fevereiro de 2022. Diário Oficial da União: Seção 1, Brasília, DF, 10 fev. 2022.
2. BRASIL. Ministério da Saúde. Portaria de Consolidação nº 1, de 22 de fevereiro de 2022. Diário Oficial da União: Seção 1, Brasília, DF, 23 fev. 2022.
3. ABCFARMA - Associação Brasileira das Indústrias de Medicamentos. Anuário Estatístico do Mercado Farmacêutico 2022. São Paulo: ABCFARMA; 2022.
4. BRASIL. Ministério da Saúde, Fundação Oswaldo Cruz, Agência Nacional de Vigilância Sanitária. Protocolo de Segurança na Prescrição, Uso e Administração de Medicamentos. Brasília: Ministério da Saúde; 2020.
5. BRASIL. Ministério da Saúde, Fundação Oswaldo Cruz, Agência Nacional de Vigilância Sanitária. Protocolo de Segurança na Prescrição, Uso e Administração de Medicamentos. Brasília: Ministério da Saúde; 2020.
6. BRASIL. Ministério da Saúde; Agência Nacional de Vigilância Sanitária. Protocolo de segurança na prescrição, uso e administração de medicamentos. Brasília: Ministério da Saúde, 2013.
7. ARRAIS, Paulo Sérgio Dourado; BARRETO, Maurício Lima; COELHO, Helena Lutéscia Luna. Aspectos dos processos de prescrição e dispensação de medicamentos na percepção do paciente: estudo de base populacional em Fortaleza, Ceará, Brasil. Cadernos de Saúde Pública, v. 23, p. 927–937, 2007.
8. BENETOLI, A. et al. Dangerous abbreviations and inadequate dose designations in drug prescriptions. Revista Brasileira de Farmácia Hospitalar e Serviços de Saúde [Internet], v. 2, n. 1, 2011.
9. DA SILVA, Gean Carlos Alves; PASCOA, Henrique; GAMA, Aline Rodrigues. Riscos e problemas relacionados aos erros de prescrições médicas: uma revisão bibliográfica. Saúde & Ciência em Ação, v. 6, n. 2, p. 134–148, 2020.
10. DASILVA PORTELA, Alyne et al. Medical prescriptions: adequate orientation to medicine use? Ciência & Saúde Coletiva, v. 15, p. 3523, 2010.
11. DE OLIVEIRA, Richardson Lemos et al. A interpretação da prescrição sob a ótica do paciente idoso analfabeto funcional e de baixa escolaridade. Research, Society and Development, v. 10, n. 2, p. e25410212494–e25410212494, 2021.
12. BRASIL. Governo do Distrito Federal. Segurança do Paciente: prescrição, uso e administração de medicamentos. [S.d.].
13. WORLD HEALTH ORGANIZATION. Global burden of preventable medication-related harm in health care: a systematic review. 2024a.
14. WORLD HEALTH ORGANIZATION. Medication safety in high-risk situations. [S.l.]: World Health Organization, 2019a.
15. WORLD HEALTH ORGANIZATION. Medication safety in polypharmacy: technical report. [S.l.]: World Health Organization, 2019b.
16. WORLD HEALTH ORGANIZATION. Medication without harm: policy brief. [S.l.]: World Health Organization, 2024b.

17. WORLD HEALTH ORGANIZATION. Medication without harm. [S.l.]: World Health Organization, 2017.
18. WORLD HEALTH ORGANIZATION. Reporting and learning systems for medication errors: the role of pharmacovigilance centres. 2014.
19. WORLD HEALTH ORGANIZATION. WHO patient safety curriculum guide: multi-professional edition. Geneva: WHO; 2011 [cited 2013 May 27]. [S.l: s.n.], [S.d.].
20. PERES, Frederico. Alfabetização, letramento ou literacia em saúde? Traduzindo e aplicando o conceito de health literacy no Brasil. Ciência & Saúde Coletiva, v. 28, p. 1563–1573, 2023.
21. REZENDE, Luís Henrique Oliveira et al. Prescrição de medicamentos: uma análise para a implantação da prescrição eletrônica ambulatorial. Revista Eletrônica Acervo Saúde, v. 12, n. 9, p. e3638–e3638, 2020.
22. SHRANK, William et al. Medication safety: Effect of content and format of prescription drug labels on readability, understanding, and medication use: A systematic review. Annals of Pharmacotherapy, v. 41, n. 5, p. 783–801, 2007.

Pamphlets and booklets with guidance for patients, accessed on May-2024:

1. National Council on Patient Education and Information. U.S. Food and Drug Administration, Center for Drug Evaluation and Research. 10 Questions to Ask About the Medicines You Take. Needy Meds Be Med Wise. USA. Access: https://www.bemedwise.org/
2. National Council on Patient Education and Information. U.S. Food and Drug Administration, Center for Drug Evaluation and Research. Use Over-the-Counter Medicines Wisely. USA. Access: https://www.bemedwise.org/
3. National Council on Patient Education and Information. U.S. Food and Drug Administration, Center for Drug Evaluation and Research. Medication Use Safety Training for SeniorsTM and MUST for SeniorsTM. BeMedWise Program at NeedyMeds. 2019. USA. Access: https://www.bemedwise.org/
4. National Council on Patient Education and Information. U.S. Food and Drug Administration, Center for Drug Evaluation and Research. JUGGLING MULTIPLE MedicatIons? What You Can Do to Stay Safe.. BeMedWise Program at NeedyMeds. 2022. USA. Access: https://www.bemedwise.org/
5. National Council on Patient Education and Information. U.S. Food and Drug Administration, Center for Drug Evaluation and Research. 6 Ways to Help Your Older Parents Use Medications the Right Way and Prevent Problems. BeMedWise Program at NeedyMeds. 2019. USA. Access: https://www.bemedwise.org/
6. Agency for Healthcare Research and Quality. Blood Thinner Pills: Your Guide to Using. Them Safely. USA. Access: www.ahrq.gov/btpills.htm.
7. United States Drug Enforcement Administration. DO'S and DON'TS of medicine disposal. USA. Access: https://www.dea.gov/ KnowYourOTC.org/safe-disposal.
8. Food and Drug Administration. Drug Disposal Options Do you have medicine you want to get rid of? USA. Access: www.fda.gov.
9. Food and Drug Administration. Medicine Safety Modeled after the FDA’s Medicines in My Home program For Families. USA. Access: scholastic.com/OTCliteracy/parents.
10. Food and Drug Administration. Think It Trough. USA. Access: web site at www.fda.gov/cder.
11. NHS Foundation Trust. How to take liquid medicine (Easy Read). Ref: 2020ER0048. © GOSH NHS Foundation Trust, April 2020 Symbols Text © Widgit Software 2002-2012. UK. Access: www.widgit-health.com
12. NHS Foundation Trust. How to take liquid medicine (Easy Read). Ref: 2020ER0048. © GOSH NHS Foundation Trust, April 2020 Symbols Text © Widgit Software 2002-2012. UK. Access: www.widgit-health.com

13. NHS Foundation Trust. NHS Foundation Trust. How to take liquid medicine (Easy Read). Ref: 2020ER0048. © GOSH NHS Foundation Trust, April 2020 Symbols © Widgit Software 2002-2012. UK. Access: www.widgit-health.com. Ref: 2020ER0049 Text © GOSH NHS Foundation Trust, April 2020 Symbols © Widgit Software 2002-2012. UK. Access: www.widgit-health.com
14. NHS Foundation Trust. How to use a suppository (Easy Read). Ref: 2020ER0331. Text © GOSH NHS Foundation Trust, April 2020 Symbols © Widgit Software 2002-2012. UK. Access: www.widgit-health.com
15. NHS Foundation Trust. How to use an inhaler (Easy Read). Ref: 2020ER0330 Text © GOSH NHS Foundation Trust, April 2020 Symbols © Widgit Software 2002-2012 UK. Access: www.widgit-health.com
16. NHS Foundation Trust. How to use ear drops (Easy Read). Ref: 2020ER0050 Text © GOSH NHS Foundation Trust, April 2020 Symbols © Widgit Software 2002-2012 (www.widgit-health.com) UK. Access: www.widgit-health.com
17. NHS Foundation Trust. How to use eye drops (Easy Read). Ref: 2020ER0047 Text © GOSH NHS Foundation Trust, April 2020 Symbols © Widgit Software 2002-2012. UK. Access: www.widgit-health.com
18. NHS Foundation Trust. How to use nose drops (Easy Read). Ref: 2020ER0048 Text © GOSH NHS Foundation Trust, April 2020 Symbols © Widgit Software 2002-2012. UK. Access: www.widgit-health.com
19. State of Victoria, Australia. Better Health Channel. Safe medication use. AU. Access: https://www.betterhealth.vic.gov.au/about/terms-of-use
20. Australian Government. Department of Health and Age Care. Updated 23 June 2022. Safe use of medicines. AU. Access: Home > Topics > Medicines

Supplementary file 2 - DATASET

Additional PDF

Supplementary file 3 - Software Mockup and Testing

To facilitate testing our prescriptions, a software mockup was created, integrating our dataset with the prompts to GPT-4.[1] This mockup presented each scenario using a simulated patient's name, age, gender, and education assigned and utilized EHR data entries to generate patient-specific instructions automatically. Our analysis involved comparing three distinct prompts (see Table 1) : (i) prompt 1 - a generic zero-shot command (a general rule) to generate instructions for taking a medication; (ii) prompt 2 - a zero-shot prompt with specialized rules tailored to each patient; (iii) prompt 3 - an enhanced zero shot prompt aimed at improvements to mitigate bias in the text and ensure more neutral, inclusive, and respectful language.[2] We compared the results achieved by GPT-4 with those obtained by Llama 3 using prompt 3.[3] We prompted LLMs using a script in Python. We performed three rounds of random cross-validations among three physicians, to ensure that the model's output met the requirements of the particular language expected to be adequate within the context in which the model will be used, involving our preset minimum requirements for comprehensive and safe directions to the patients.[4]

References

1 OpenAI, Achiam J, Adler S, *et al.* GPT-4 Technical Report. 2024; published online March 4. http://arxiv.org/abs/2303.08774 (accessed Sept 11, 2024).

2 Raza S, Bamgbose O, Ghuge S, Reji DJ. Safe and Responsible Large Language Model Development. 2024; published online May 21. DOI:10.48550/arXiv.2404.01399.

3 Dubey A, Jauhri A, Pandey A, *et al.* The Llama 3 Herd of Models. 2024; published online Aug 15. http://arxiv.org/abs/2407.21783 (accessed Sept 11, 2024).

4 Reis Z, Pagano AS, Lage E, *et al.* SOP for personalized directions in e-prescription. 2024; published online June 17. https://www.protocols.io/view/sop-for-personalized-directions-in-e-prescription-dckh2ut6 (accessed June 17, 2024).

Supplementary file 4 - Assessment-tool of the model's output

Guidelines for evaluators: Think of a real consultation in a primary care scenario to assess the model's generated text. You may decide to use the AI-generated text in its entirety or improve it by editing in the box below as you would think it most appropriate to deliver it to your patient.

1 - How would you evaluate the adequacy of the AI-generated instructions compared to the reference text? (Scale from 1 to 5):

- Fully adequate
- Partially adequate
- Neither adequate nor inadequate
- Partially inadequate
- Completely inadequate

Please justify your answer.

2 - How would you evaluate the acceptability (clarity and text flow) of the AI-generated instructions compared to the reference text? (Scale from 1 to 5):

- Fully acceptable
- Partially acceptable
- Neither acceptable nor unacceptable
- Partially unacceptable
- Completely unacceptable

Please justify your answer.

3 - How would you evaluate the AI-generated instructions in terms of personalization (age, education, gender, reason for consultation)? (Scale from 1 to 5):

- Fully satisfactory
- Partially satisfactory
- Neither satisfactory nor unsatisfactory
- Partially unsatisfactory
- Completely unsatisfactory

Please justify your answer.

4 - Error analysis: Please select all the categories that apply to the errors found. If no error was found, leave it blank.

Type 1: LLM provided instruction capable of causing people to adhere to incorrect medication use

Type 2: LLM provided instruction containing vague arguments or unsupported conclusions

Type 3: LLM ignored essential information on the use of the medicine

Type 4: LLM provided instruction containing factual, non-medical error (incorrect mathematical operations

or language use ascribable to LLM translation into Portuguese)

Type 5: LLM provided instruction unsupported by scientific evidence

Type 6: LLM provided instruction unexpected for the requested task

Type 7: LLM provided instruction containing incorrect information or hallucinations

Supplementary File 5. Sample of LLM output errors

Type of error	Generated instructions<br>(original Portuguese with English gloss)	Explanation
LLM provided instruction capable of causing people to adhere to incorrect medication use	Lucas, você deve tomar o medicamento Cefalexina, que é uma cápsula de 500 mg. A forma correta de tomar é a seguinte: ingira 1 cápsula a cada 6 horas. Para facilitar, você pode associar a tomada do medicamento às suas refeições principais e antes de dormir. Lembre-se de tomar a cápsula com um copo cheio de água, olhando para baixo e colocando o remédio em cima da língua, mais perto da garganta.<br><br>*Lucas, you must take the drug Cephalexin, which is a 500 mg capsule. The correct way to take it is as follows: take 1 capsule every 6 hours. To make it easier, you can take the medication together with the main meals you have and before going to bed. Remember to take the capsule with a full glass of water, looking down as you swallow and placing the medicine on the back of your tongue, closer to your throat.*	Text instructs the patient to take an antibiotic at flexible times, when antibiotics are expected to be taken in a strict regime, at the same time everyday.
LLM provided instruction containing vague arguments or unsupported conclusions	O tratamento deve durar 7 dias, totalizando 28 cápsulas. Portanto, você deve tomar o medicamento até o fim, mesmo que os sintomas da infecção na pele já tenham desaparecido antes desse período. Antes de cada uso, confira o nome do medicamento e a concentração da dose na embalagem. Mantenha o medicamento em sua embalagem original e guarde-o em um local seguro, fora do alcance de crianças.<br><br>*The treatment lasts 7 days, with 28 capsules in total. Therefore, you must take the medication until it is finished, even if the symptoms of your skin infection have cleared up before that period. Before each use, check the name of the medication and the dose concentration on the packaging. Keep the medication in its original packaging and store it in a safe place out of children's reach.*	Text shows an incorrect logical marker ("therefore") linking two unconnected ideas.
	Ricardo, você deve tomar o medicamento Anlodipino, Besilato de 5 mg, que é um comprimido. A forma correta de tomar é uma vez ao dia. Você pode escolher um horário que seja mais conveniente para você, mas é importante que seja sempre no mesmo horário todos os dias. Por exemplo, se escolher tomar às 8h da manhã, deve tomar todos os dias às 8h da manhã.<br>Este medicamento deve ser engolido inteiro, sem mastigar ou partir, e pode ser tomado com um copo de água. Não é necessário estar em jejum para tomar este medicamento, você pode tomá-lo antes, durante ou após as refeições. O tratamento é por tempo indeterminado, ou seja, você deve continuar tomando todos os dias, sem interrupção.<br><br>*Ricardo, you must take the medication Anlodipine, Besylate 5 mg, which is a tablet. The correct way to take it is once a day. You can choose a time that is most convenient for you, but it is important that you always take it at the same time every day. For example, if you choose to take it at 8am, you should take it at 8 a.m. every day. This medication should be swallowed whole, without chewing or opening it, and can be taken with a glass of water. You don't need to fast to take this medication, you can take it before, during or after meals. The treatment is for an unlimited period, i.e. you should continue taking it every day without interruption.*	Text provides contradicting instructions: taking the medicine at 8 a.m. in the morning every day versus taking the medication either before, during or after meals.

LLM ignored essential information on the use of the medication	Lívia, você deve usar o esmalte Amorolfina, Cloridrato 50 mg/ml. Aplique uma camada do esmalte diretamente nas unhas afetadas, uma vez por semana. Antes de aplicar, limpe e seque bem as unhas. Use o pincel que acompanha o frasco para aplicar o esmalte. É importante cobrir toda a área afetada da unha.<br><br>*Livia, you must use the nail polish Amorolfine Hydrochloride 50 mg/ml. Apply a layer of nail polish directly onto the affected nails once a week. Before applying, clean and dry your nails thoroughly. Use the brush that comes with the bottle to apply the nail polish. It is important that you apply it to the entire affected area of the nail.*	Text provides no instruction as to how to clean the nail using isopropyl alcohol, which is included in the medication package
LLM provided instruction containing factual, non-medical errors (incorrect mathematical operations or language use ascribable to LLM translation into Portuguese)	Juliana, você deve tomar o medicamento Valaciclovir, Cloridrato 500 mg Comprimido. Tome 2 comprimidos a cada 8 horas. É importante tomar os comprimidos com um copo de água para ajudar a engolir. Continue este tratamento por 7 dias.<br>Certifique-se de tomar os comprimidos nos horários corretos: um pela manhã, outro à tarde e o último à noite, para manter o intervalo de 8 horas entre cada dose. Tome este medicamento todos os dias. Antes de acabar os que você recebeu, retorne na unidade de saúde para receber uma nova receita. Mantenha os comprimidos em sua embalagem original e guarde-os em um local seguro, fora do alcance de crianças.<br><br>*Juliana, you must take the medication Valaciclovir, Hydrochloride 500 mg Tablets. Take 2 tablets every 8 hours. It is important that you take the tablets with a glass of water to aid swallowing. Continue this treatment for 7 days.*<br>*Make sure you take the tablets at the correct times: one in the morning, one in the afternoon and one in the evening, to maintain the 8-hour interval between each dose. Take this medication every day. Before you run out of the pills you have been supplied with, make an appointment at your health center to get a new prescription. Keep the tablets in their original packaging and store them in a safe place out of children's reach.*	Text provides wrong computation of medication dosage: it instructs the patient to take two tablets every 8 hours and at the same time to take one tablet in the morning, another one in the afternoon and another one in the evening.
LLM provided instruction unsupported by scientific evidence	Como Aplicar:<br>- Antes de aplicar o creme, lave bem as mãos e a área afetada com água morna e um sabonete suave. Seque suavemente com uma toalha limpa.<br><br>*How to apply*<br>*- Before applying the cream, wash your hands and the affected area thoroughly with warm water and a mild soap. Dry gently with a clean towel.*	Texts provides instructions clinically unsupported: there is no need to use warm water or a mild soap to wash the skin
LLM provided instruction unexpected for the requested task	Importância do Ácido Fólico na Gravidez: O ácido fólico é muito importante durante a gravidez, pois ajuda a prevenir defeitos no tubo neural do bebê, que é a estrutura que dá origem ao cérebro e à medula espinhal.<br><br>*Importance of Folic Acid in Pregnancy: Folic acid is very important during pregnancy as it helps prevent defects in the baby's neural tube, which is the structure that develops into the brain and spinal cord.*	Text is not expected as part of instructions on how to take a medication
LLM provided instruction containing	Adesão ao tratamento: É crucial tomar o medicamento exatamente como prescrito, sem pular doses, mesmo que você comece a se sentir melhor. Parar o tratamento precoce pode fazer com que a infecção não seja completamente curada e pode aumentar o risco de resistência ao medicamento.	Text provides an incorrect explanation about the specific

incorrect information or hallucinations	*Treatment adherence: It is important that you take the medication exactly as prescribed, without skipping doses, even if you start to feel better. Stopping treatment early can result in the infection not being completely cleared and can increase the risk of medication resistance.*	antibiotic being prescribed. Spiramycin decreases the chance that the unborn baby will get the infection.

Supplementary file 5. Sample error-free LLM output

Acute disease	Ana Luiza, tome o remédio chamado Amoxicilina + Clavulanato de Potássio, que tem 875 mg + 125 mg, a cada 12 horas. Isso significa que você deve tomar 1 comprimido 2 vezes ao dia, por exemplo, tome 1 comprimido às 8 horas da manhã e 1 comprimido às 8 horas da noite. Retire o comprimido da embalagem apenas quando for tomar. Olhando para baixo, coloque o comprimido em cima da língua. Tome o comprimido com água para facilitar engolir o comprimido inteiro, de preferência quando puder comer algum alimento em seguida.<br>São 14 comprimidos no total. Continue tomando o remédio por 7 dias seguidos. Mesmo que você se sinta melhor antes, não pare de tomar o remédio até acabar todos os comprimidos. Não compartilhe e nem ofereça esse comprimido para outra pessoa. Cuide para que os comprimidos fiquem longe do alcance de crianças.<br><br>*Ana Luiza, take the medication called Amoxicillin + Clavulanate Potassium, which contains 875 mg + 125 mg, every 12 hours. This means that you must take 1 tablet twice a day, for example, take 1 tablet at 8 o'clock in the morning and 1 tablet at 8 o'clock in the evening. Remove the tablet from the package only when you are ready to take it. Looking down, place the tablet on the back of your tongue. Take the tablet with water to make it easier to swallow the whole tablet, preferably when you can have something to eat afterwards.*<br>*There are 14 tablets in total. Continue taking the medication for 7 days continuously. Even if you feel better earlier, don't stop taking the medication until you've finished all the tablets. Do not share or give this pill to someone else. Make sure that the tablets are kept out of children's reach.*
Chronic disease	Larissa, tome 1 comprimido de Rivaroxabana de 20 mg por dia, sempre no mesmo horário, todos os dias. É importante que você tome o comprimido junto com a comida, pode ser no café da manhã, por exemplo. Use água para engolir o comprimido inteiro, sem partir ou mastigar. Mantenha os comprimidos na embalagem original para protegê-los da umidade e do excesso de calor.<br>O tratamento é só para você. Não empreste e nem compartilhe esse medicamento. Retorne ao serviço de saúde para buscar nova receita antes de terminar os 120 comprimidos.<br><br>*Larissa, take 1 tablet of Rivaroxaban 20 mg a day, always at the same time, every day. It is important that you take the tablet with some food, for example at breakfast. Use water to swallow the tablet whole, without crushing or chewing. Keep the tablets in their original packaging to protect them from moisture and excess heat.*<br>*The treatment is for you alone. Do not lend or share this medication. Make an appointment at your health center for a new prescription before you have finished the 120 tablets.*

Supplementary file 6. Outcomes and Statistics with details

Primary Outcome, The primary outcome of this study was a statistical analysis of the dependent comparisons among the scores from 104 evaluations conducted by physicians in three rounds. These evaluations assessed the quality of GPT-4 outputs generated from three distinct prompts, namely prompt 1, prompt 2, and prompt 3, against a human-generated reference text. Next, we compared GPT-4 results for prompt 3 and Llama 3 results. Categorical variables were analyzed in terms of frequency and presented with 95% Confidence Intervals (CIs), while numerical variables were described using measures of central tendency (mean, median) and variability (standard deviation, range), adhering to their parametric frequency distribution.

To analyze the dependent quantitative data, a General Linear Model was utilized to compare the mean scores across the three prompts. Subsequent pairwise comparisons between the groups were performed using the Bonferroni correction to adjust for multiple testing. A paired T-mean test compared similarity values between GPT-4 and Llama 3 generated output against our reference texts. The Chi-square McNemar-Bowker Test was applied to evaluate marginal homogeneity among the categories, assessing the comparative performance between each pair of prompts (prompt 1 vs. prompt 2, prompt 1 vs. prompt 3, prompt 2 vs. prompt 3, and GPT-4 vs. Llama 3 for prompt 3) throughout the progression of prompts. Pearson's Chi-square Test was employed to analyze the independence of categorical frequencies. P-values less than 0.05 were considered statistically significant. Proportions and mean differences were reported alongside their 95% CIs. All statistical analyses were conducted using SPSS software (version 26.0; IBM Corp).

Secondary Outcome The secondary outcome involved using vector representations (word embeddings) to compare the outputs of the LLMs (GPT-4 and Llama 3) with reference texts, aiming to evaluate the semantic proximity between the sentences. The sentences are converted into vectors using OpenAI's text-embedding-ada-002 model, a dense and contextual representation, which transforms each sentence into a vector of fixed dimensions. The embeddings, high-dimensional vector representations, capture the semantic meanings and relationships between words in a sentence, sentences close to each other in this space tending to share similar meanings. The similarity between the representative vector of each reference text and its

counterpart generated by the LLM from one of the three proposed prompts is calculated. We computed the average similarity between the 104 pairs (reference text and generated text) for each prompt, reporting the average value and the corresponding standard deviation.

By transforming words into vector representations, the algorithm processes linguistic information, facilitating an understanding of semantic relationships and contextual meanings. Each sentence is converted into a vector that encapsulates its semantic content, and the algorithm evaluates the proximity between these vectors to determine textual similarity. The results of this analysis are presented as percentages, indicating the degree of similarity between the texts generated by the three prompts and the reference text for each clinical scenario. As we focus on evaluating semantic similarity, we consider the cosine similarity metric. More specifically, we adapt this metric to report a value between 0 and 100% to facilitate interpretation. The literature reports that cosine similarity outperforms other metrics for semantic evaluation. Moreover, cosine similarity is less sensitive to differences in text size.[1]

References

1 Kumawat T. Empowering Natural Language Processing with OpenAI Embeddings: Text Similarity, Semantic Search…. Medium. 2023; published online June 11. https://medium.com/@tejpal.abhyuday/empowering-natural-language-processing-with-openai-embeddings-text-similarity-semantic-search-71617867760f (accessed June 9, 2024).
